# Ablation manual for liver cancer

**DOI:** 10.1007/s10396-024-01499-9

**Published:** 2024-10-12

**Authors:** Hitoshi Maruyama, Yasunori Minami, Katsutoshi Sugimoto, Akihiro Funaoka, Kazushi Numata

**Affiliations:** 1 Department of Gastroenterology, Juntendo University, 2-1-1 Hongo, Bunkyo, Tokyo, 113-8421 Japan; 2 Department of Gastroenterology, Faculty of Medicine, Kindai University, Ohno-Higashi Osaka-Sayama, Osaka, 589-8511 Japan; 3 Department of Gastroenterology and Hepatology, Tokyo Medical University, 6-1-1 Shinjuku, Tokyo, 160-8402 Japan; 4 Gastroenterological Center, Yokohama City University Medical Center, 4-57 Urafunecho, Minami, Yokohama, Kanagawa 232-0024 Japan

**Keywords:** Ablation, Ultrasound, Hepatocellular carcinoma, Liver, Cancer

## Abstract

Because of recent advances in energy device technology, ablation has become popular worldwide. It is less invasive and provides faster postoperative recovery compared to surgery, and therefore, it has come to be applied to a wide range of organs, such as liver, lung, kidney, thyroid, and bone/soft tissue tumors. In order to properly guide the needle to the target area, imaging support is necessary, and ultrasound, which has the advantages of high resolution and real-time capability, is the most frequently used modality. In other words, ablation can be said to be a therapeutic method that makes the most of the advantages of ultrasound. This article outlines the role of ultrasound in ablation for liver cancer and its specific usage.

## Introduction

Radiofrequency ablation (RFA), microwave ablation (MWA), cryoablation, and ethanol infusion therapy are collectively referred to as ablation. RFA and MWA, which are techniques for achieving a tumor necrosis effect by means of heat, are regarded as radical treatment, like surgery [1–3]. They are generally performed under intravenous anesthesia or local anesthesia, with the applicator typically being guided to the target site using ultrasound (US). An antitumor effect is achieved via ablation over a certain area from the applicator tip, which is less invasive than open surgery. They are often used to treat primary/metastatic liver cancer, but they are also performed for lesions in lung, bone, kidney, thyroid gland, adrenal gland, lymph nodes, and so on. Although not currently covered by insurance, promising results have been reported for ablation of non-malignant hepatic tumors such as hepatic hemangioma and hepatic adenoma [4].

## Principles of ablation (RFA, MWA)

In an environment that exceeds 50 °C, biological tissue undergoes cell death as a result of injury to the cell membrane and mitochondria, and due to thermal denaturation of various enzymes. RFA and MWA are techniques that induce a tumor necrosis effect via such high temperatures.

RFA necroses cancer tissue by using high-frequency waves (about 450 kHz) to create Joule heat around an electrode. In addition, increased temperatures due to heat transfer are seen in areas some distance from the electrode. In the case of a single-needle electrode, perfusion of cooling water suppresses the rise in the temperature of the electrode needle and the surrounding area and expands the ablation area. The impedance increases when the temperature around the electrode exceeds 100 °C and the moisture in the tissue evaporates. This results in a state in which an electric current is not generated and thermal energy is not supplied, which is referred to as roll-off. When this happens, powering down of the device causes moisture to move from surrounding tissue to the area where the moisture had evaporated, allowing it to be powered up again. Roll-off is used as a measure for the ablative effect of RFA treatment, and it is believed that treatment should be completed after at least one roll-off has occurred.

RFA devices currently available in Japan and their features are shown in Table 1. Since most of the RFA devices available in Japan are non-deployable, single-needle devices, this review article will focus on non-deployable, single-needle devices.

**Table 1:** Features of radiofrequency ablation systems available in Japan

Models	Cool-tip	VIVA	arfa	Celon Power	LeVeen
Japanese vendors	Covidien Japan	Century Medical	Japan Lifeline	Olympus Medical Systems	Boston Scientific Japan
Configuration of electrode needle	1 non-deployable needle	1 non-deployable needle	1 non-deployable needle	1–3 non-deployable needles	1 deployable needle
Features	The indications have been expanded to the following disorders: Small renal malignancy Pulmonary malignancy Malignant bone tumor Osteoid osteoma Intrapelvic malignancy Soft tissue tumor in extremities, thoracic cavity, and abdominal cavity Hemostasis for acardius in acardiac twins	Length of non-insulated part of electrode tip can be adjusted Electrode diameter comes in 15 G, 17 G, and 18 G variations	Length of non-insulated part of electrode tip can be adjusted Equipped with two temperature sensors, one for the tip and one for water temperature The only model manufactured in Japan	The only model covered by health insurance for adrenal tumors The only bipolar current type	

MWA is a technique to provide coagulative necrosis by generating heat via irradiation with high-frequency (about 2450 MHz) microwaves. It also uses a water cooling system that can control the temperature of a needle-shaped applicator (antenna) and result in a stable ablative effect. The demand for MWA is growing as it can produce an ablative effect larger than that of RFA.

## Summary of ablation for hepatocellular carcinoma (HCC)

In the case of ablation of HCC, it is important that the ablation includes not only the tumor itself but also the adjacent area (generally an ablative margin of 5 mm) in order to avoid residual tumor and local recurrence [5]. Therefore, treatment must be performed while three-dimensionally visualizing the contour of the tumor. On the other hand, attention must also be paid to hepatic reserve during treatment as many patients with HCC have concomitant liver cirrhosis. In other words, it is important to both attempt to achieve total necrosis of the cancer including the area adjacent to the tumor and avoid excessively affecting the surrounding non-tumorous liver parenchyma.

B-mode US is basically used during the entire process from preoperative planning, making artificial pleural effusion and/or artificial ascites and other supporting procedures, puncture, and monitoring during ablation to needle removal. In addition, assistive technologies such as color Doppler, fusion imaging, and contrast-enhanced US are effectively utilized for safe and effective ablation.

US is generally the diagnostic imaging modality most frequently used in patients with liver disease, but ethoxybenzyl diethylenetriamine pentaacetic acid-enhanced magnetic resonance imaging (EOB-MRI) and contrast-enhanced computed tomography (CT) are often used for surveillance of patients at risk for HCC. However, identification of a mass using US is not necessarily easy even in cases where HCC has been presumptively detected on MRI or CT. This is because liver atrophy/deformation and coarsened parenchymal echo associated with coexisting liver cirrhosis and other factors work against identification of the tumor. In addition, many recurrent patients with a history of treatment such as surgery, ablation, and catheter treatment are encountered, and the prior treatment often makes it difficult to recognize the treatment target site. In such cases, assistive technologies like fusion imaging and contrast-enhanced US are extremely useful for identifying the tumor on US and completing the ablation.

The results of a prospective clinical study conducted in Japan to compare ablation and surgery were published recently. The study, called the SURF trial, was a multicenter, randomized, comparison study (comparison of hepatectomy and RFA) in primary HCC patients (Child–Pugh score ≤ 7) with largest diameter ≤ 3 cm and ≤ 3 nodules [6]. A significant difference was not found between the RFA group and the surgical resection group in terms of 5-year recurrence-free survival and overall survival, negating the superiority of surgery. Based on this finding, ablation is now indicated for Child A/B HCC with a diameter ≤ 3 cm/ ≤ 3 nodules and without vascular invasion/distant metastasis, and it is now ranked the same as surgery, in the latest Clinical Practice Guidelines for Hepatocellular Carcinoma 2021 [7].

## Ablation-related technologies/procedures

### Preoperative planning

This corresponds to simulation of the puncture procedure, and it should be performed the day before treatment whenever possible. It is important to evaluate the patient before treatment as one may find occurrence of ascites or an increase in tumor size (including occurrence of portal vein tumor thrombus, etc.) not seen when scheduling hospitalization in the outpatient clinic. In addition, evaluation with US allows one to evaluate the patient's general condition that cannot be ascertained based on hematological findings, with US being mandatory not only for beginners but also those experienced in ablative therapy.

**Fig. 1:**
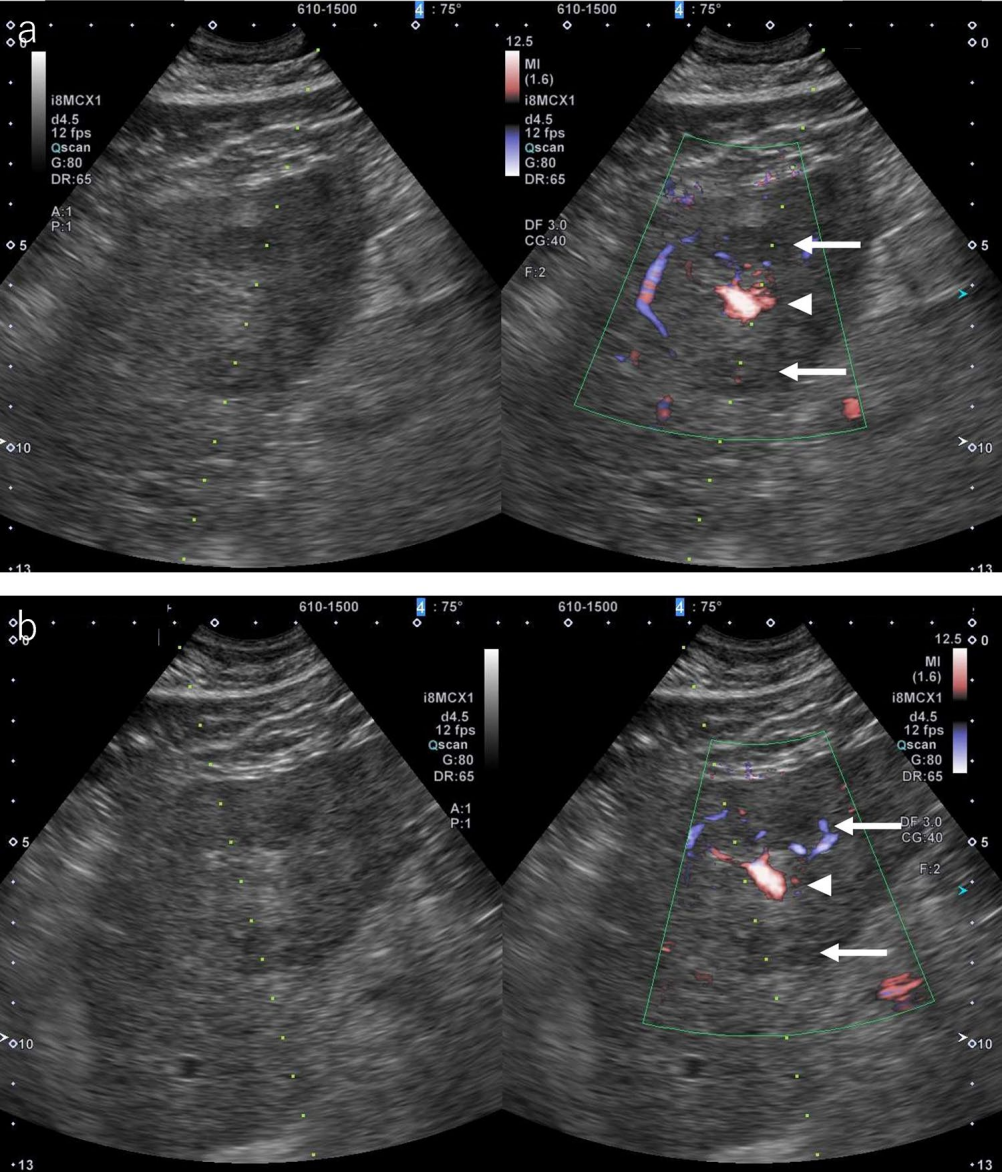
Planning. **a** HCC is found in two places in S3 of the liver. It is located ventrally in P3 and dorsally, respectively (arrows). It is depicted more clearly in P3 (arrowhead) when color Doppler is used. The dorsal lesion was to be ablated first, but it was determined that it was not appropriate as P3 was located on the puncture route when the puncture line was in the forward direction. **b** P3 could be circumvented from the puncture route by planning a puncture line in the opposite direction

During planning, the operator needs to demonstrate the puncture needle insertion route from the skin/liver surface to the tumor using B-mode US. It is important to use the same probe that will be used during the actual treatment. If the tumor is located in the right hepatic lobe, it should be observed between multiple ribs to find the intercostal space that allows the tumor to be observed the most clearly. Next, assess whether there are any large vessels (particularly the portal vein) along the puncture route. It is easy to distinguish the portal vein from hepatic veins based on the anatomical course, but color Doppler US should be used when it is difficult to differentiate them. If there is a large vessel along the puncture route, consider flipping the US screen horizontally and switching to a puncture route from the reverse direction (cranial), not the forward direction (caudal) (Fig. 1a, b). When puncture is difficult due to the presence of a vessel along the puncture route, consider another route from a different intercostal space. However, a puncture route looking up from between the ribs should be avoided as there is a risk of intercostal arterial injury. In addition, a change of patient position should also be considered if necessary to gain a better puncture route. In the case of a tumor under the hepatic dome in segment 4 (S4), in particular, visualization is sometimes difficult when scanning looking up from the cardiac space, but visualization might be easier by putting the patient in the sitting position.

Depending on the site of the lesion, visualization might be difficult without using artificial ascites/pleural effusion. In such cases, deciding on the infusion site of an artificial ascites/pleural effusion beforehand will result in smoother execution of the procedure. If there are multiple lesions requiring treatment, it is important to determine beforehand the order in which they will be treated. The deepest lesion is generally treated first, but when performing treatment under artificial ascites, treatment should be performed promptly when the conditions are right as the visibility of the lesion might deteriorate due to ascites moving over time. In the case of a large lesion for which ablation needs to be performed multiple times, the order of the punctures should be decided in advance. At institutions where both RFA and MWA are available, it is best to decide at the planning stage which one should be used.

Education of those inexperienced in ablative therapy is a large part of the planning process. Beginners are often satisfied with the tumor visualization achieved by scanning with the dominant hand, but in most cases the dominant hand is used to hold the puncture needle while scanning with the probe using the opposite hand during an actual puncture. Therefore, whether a beginner can reliably depict a lesion using the non-dominant hand needs to be confirmed. Moreover, it is difficult for beginners to reliably depict a lesion due to the effects of patient respiration, but experienced operators can continue to reliably depict a lesion by fine-tuning scanning with the probe according to the patient's respiration even if the patient does not hold their breath. Furthermore, a doctor performing ablative therapy must be familiar with the acoustic features of US and how to perform contrast-enhanced US, fusion, and needle navigation. Beginners should keep these points in mind when going into planning. Depending on the institution, technicians are in charge of routine US examinations while physicians only perform US during treatment, but it is important to get used to performing US on a regular basis as US skills cannot be acquired overnight.

### Puncture probe

**Fig. 2:**
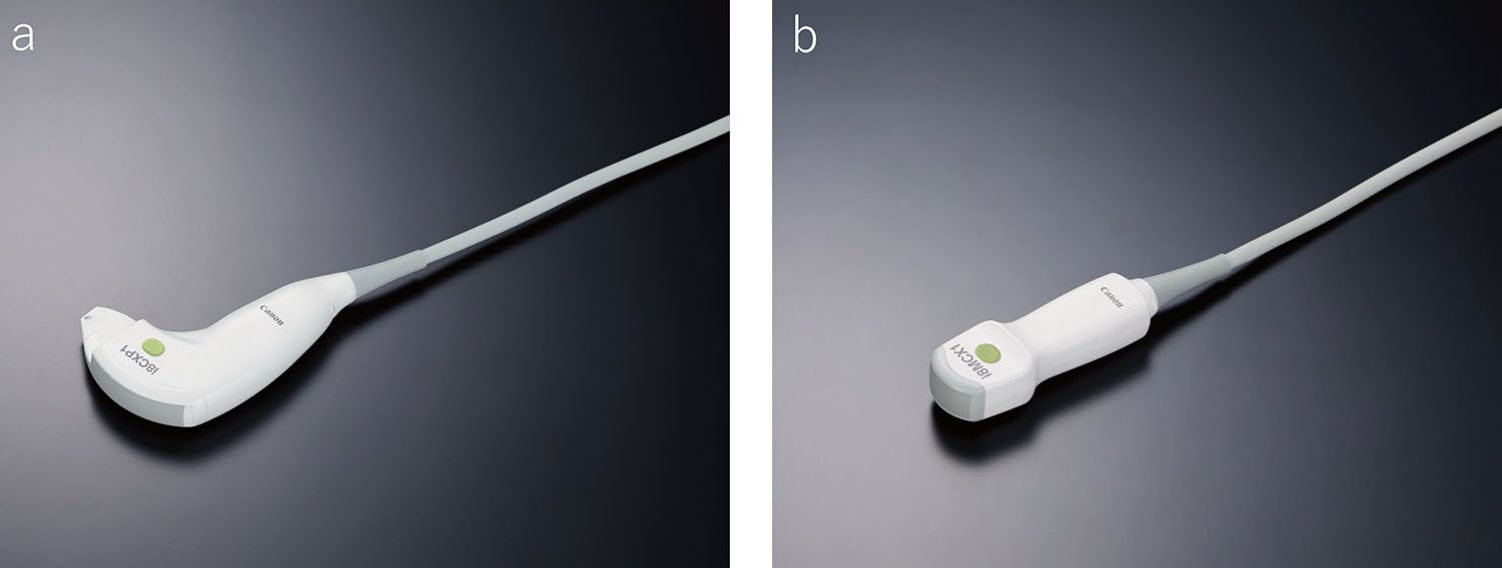
**a** Dedicated puncture probe. **b** Micro convex probe

There are two types of probes used for US-guided puncture procedures: a dedicated puncture probe that integrates the scanning surface and puncture hole (Fig. 2a) and a type that is used by fitting an attachment to a standard probe. A regular convex probe is sometimes used for the latter, but a small micro convex probe (Fig. 2b) with a small diameter developed for puncture use is often utilized. There are both advantages and disadvantages with each type of puncture probe. Some advantages of a dedicated puncture probe that integrates the scanning surface and puncture hole include the absence of a blind spot during needle puncture, the ability to puncture from an angle perpendicular to the probe surface, and image quality equivalent to that of a regular convex probe.

A micro convex probe, on the other hand, is not as good as a dedicated puncture probe or convex probe in terms of image quality, but it provides a high degree of freedom for intercostal scanning as the probe radius (R) is small and the thickness of the probe is thin. It is particularly good for depicting lesions under the hepatic dome. It should be noted that the specification/configuration and selectable puncture angles of micro convex probes differ depending on the manufacturer.

### Treatment assistive technologies

#### Concomitant use of artificial pleural effusion/ascites

Artificial pleural effusion/ascites is a very useful assistive technology used to improve the ability to visualize the tumor with US and to avoid thermal damage to areas other than the target site. The fluid infused is 5% glucose solution or physiological saline, each of which has associated complications, as described below. The former is generally used at many institutions in order to avoid conductivity.

A. Artificial pleural effusion method

**Fig. 3:**
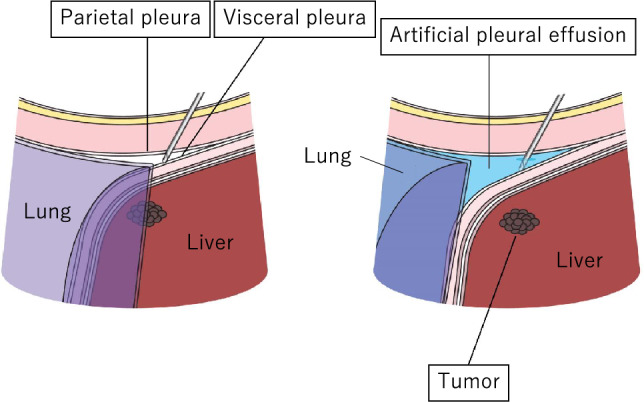
Anatomical structure that needs to be understood when creating artificial pleural effusion

By injecting fluid into the thoracic cavity, the artificial pleural effusion method allows one to depict lesions in the right hepatic lobe that are difficult to visualize with intercostal scanning under the influence of gas in the lung. There are various reports on how to create artificial pleural effusion [8–10], but pleural effusion is often created by placing a puncture needle (pneumoperitoneum needle) at a position directly above the diaphragm or that slightly pierces the diaphragm of the hepatic surface at a position slightly caudally distant from the lungs, utilizing the fact that the thoracic cavity covers the entire right hepatic lobe [11]. A schematic that shows the anatomical structure needed when creating artificial pleural effusion is shown in Fig. 3.

- Procedure

**Fig. 4:**
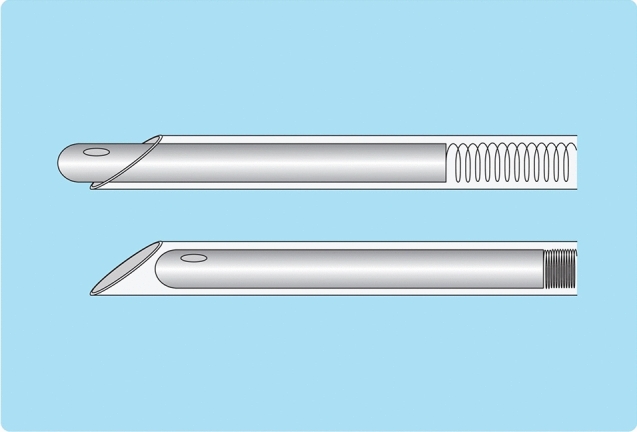
Structure of pneumoperitoneum needle

Using a 22G Cathelin needle, administer intradermal anesthesia, and then inject local anesthesia while advancing the needle under US guidance. After administering anesthesia, the risk of puncturing the liver parenchyma with the pneumoperitoneum needle can be avoided by creating space by injecting 5% glucose solution into a layer deeper than the thoracic cavity such as below the liver capsule. The pneumoperitoneum needle has a dual structure comprised of an inner needle with a dull tip and a sharp outer needle (Fig. 4). In its resting state, the inner needle protrudes from the tip of the outer needle. When the needle is pressed strongly against the pleura, the inner needle retreats and is pushed inside the outer needle, and the tip of the pneumoperitoneum needle becomes sharp, allowing the pleura to be pierced. The tip of the needle returns to the original dull state when the needle is placed in the thoracic space. There is a side hole for injecting water just before the tip of the inner needle, and stable pleural effusion can be created by slightly piercing the diaphragm. In addition, it is important to perform the puncture so that the pneumoperitoneum needle is as vertical to the liver as possible. Water may collect in subcutaneous tissue when the position of the pneumoperitoneum needle is shallow, water will collect in the diaphragm when it is slightly deep, and ascites will be created when it is even deeper. These can be easily distinguished by observing the intravenous drip rate and the status of pleural fluid injection on US (e.g., thickening of subcutaneous tissue and diaphragm). Moreover, when pleural fluid is being properly injected, respiratory fluctuations are seen in the intravenous drip. An injection volume of 500 mL is sufficient in most cases. When visualization of the lesion is still poor, the backrest of the bed should be raised further; the injection volume should not be increased. Draining the injected pleural fluid is not necessarily needed as it will usually be mostly absorbed naturally within several days, but draining it can make the patient's recovery time and hospital stay shorter as compared with waiting for it to disappear naturally in patients with low hepatic reserve and patients with coexisting lung disease.

b. Complications

Risks associated with the creation of artificial pleural effusion are subcutaneous hematoma, unintended lung puncture (pneumothorax), and intercostal arterial injury (hemothorax). Examples of side effects of the injected fluid are respiratory distress, hypoxemia, hyponatremia due to 5% glucose solution, and cardiac load due to physiological saline, but an injection volume of about 500 mL usually poses little problem. However, artificial pleural effusion is contraindicated in patients with severe chronic lung disease and patients without a left lung as hypoventilation due to artificial pleural fluid injection or complications due to pneumonia/pneumothorax/hemothorax could be fatal. There is also a case report of an unfortunate outcome due to exacerbation of interstitial pneumonia that may have been caused by pleural effusion creation [12]. In addition, since pleural effusion cannot be properly created in patients with adhesion between lung and pleura, it is important to take into account that possibility and consider a different treatment plan in patients who have undergone local ablative therapy for a lesion under the dome in the past or patients with a thickened pleura detected on preoperative CT.

B. Artificial ascites method

By injecting fluid into the abdominal cavity, the artificial ascites method allows one to depict lesions in the right hepatic lobe that are difficult to visualize with intercostal scanning under the influence of gas in the lung. It is also a useful technique for avoiding thermal injury to adjacent organs and an indispensable procedure in local ablative therapy. There are various reports on how to create artificial ascites [13], but here we will present visualization of a lesion under the right diaphragm and a method for direct puncture of the liver with a pneumoperitoneum needle designed to prevent thermal injury of the diaphragm [11]. In addition, artificial ascites is created by inserting the pneumoperitoneum needle near the lesion in order to prevent thermal injury to the stomach and large intestine as much as possible.

- Procedure

**Fig. 5:**
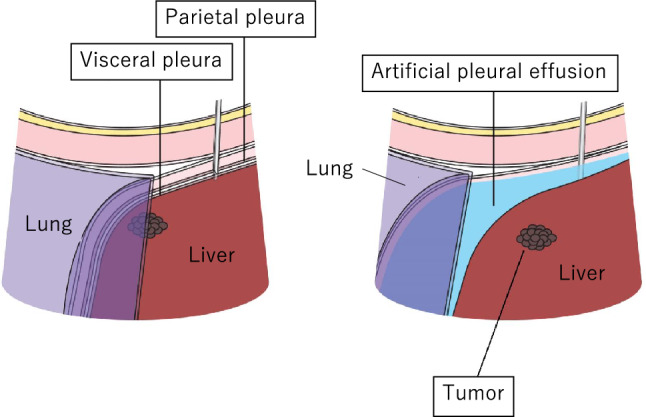
Anatomical structure that needs to be understood when creating artificial ascites

We will explain artificial ascites creation by means of direct liver puncture. The anatomical structure needed when creating artificial ascites is shown as a schematic (Fig. 5).

**Fig. 6:**
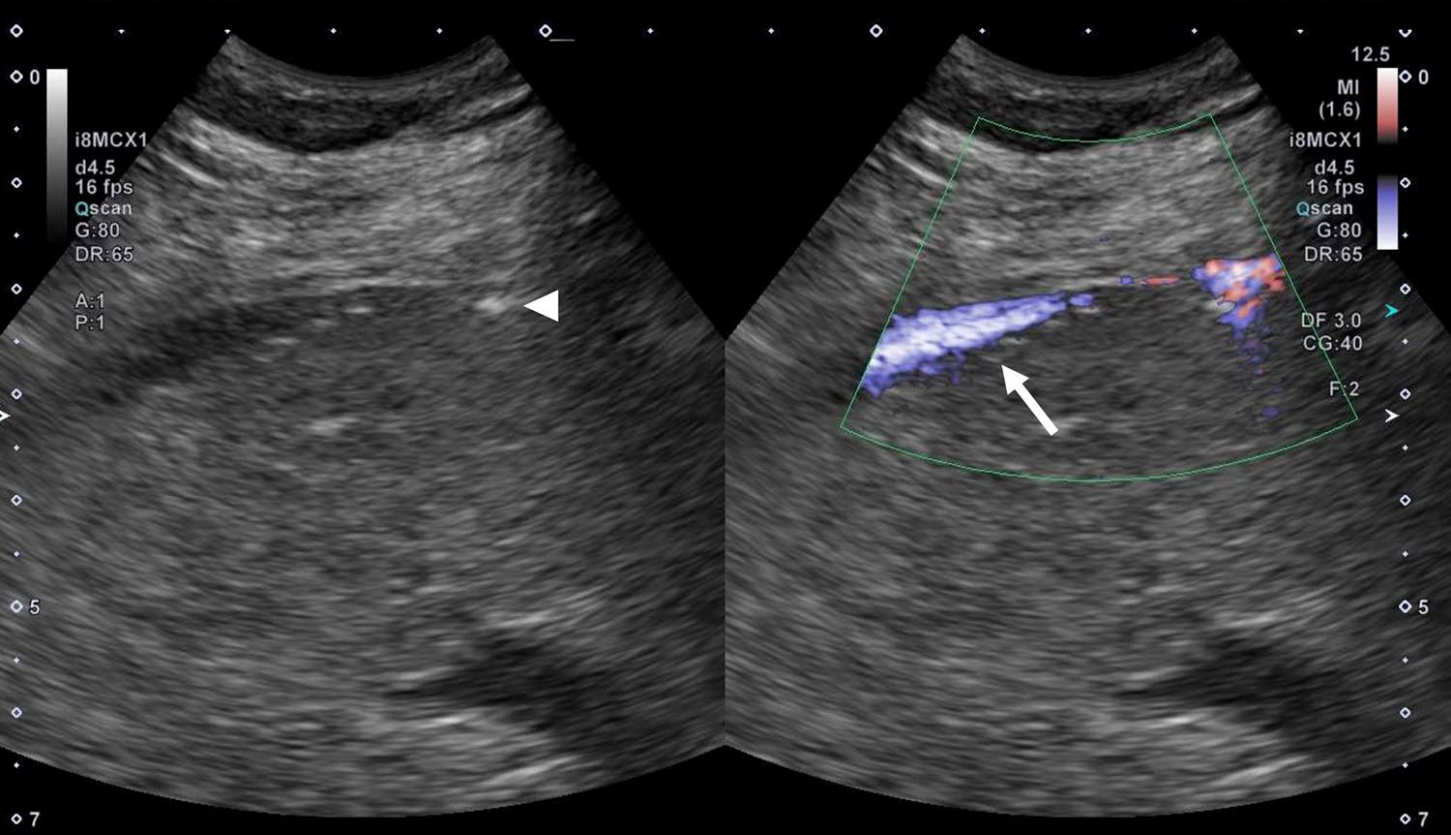
The flow of 5% glucose solution from the tip of the pneumoperitoneum needle (arrowhead) into the abdominal cavity is clearly depicted on color Doppler US (arrow)

Select the puncture site that allows clear visualization of the right hepatic lobe on right intercostal scanning. Under US guidance, administer local anesthesia to the liver capsule, subcutaneous tissue, and skin, in that order. Next, make an incision in the skin with a pointed blade and secure the puncture route with mosquito forceps. Then, perform direct puncture with the tip of the pneumoperitoneum needle to a depth of about 1 cm from the liver surface under US guidance. After proper puncture, fully open the IV set and slowly pull out the pneumoperitoneum needle while monitoring with US. When the tip of the pneumoperitoneum needle is about 5 mm from the liver surface, ascites will flow into the abdominal cavity all at once by means of hydraulic pressure. This process can also be monitored using color Doppler US (Fig. 6). When the water pools, an echo-free space is created in the abdominal cavity under the liver surface and diaphragm; therefore, more stable ascites can be created by removing the pneumoperitoneum needle from the liver and inserting it in the echo-free space. The amount of ascites injected is usually about 500–1000 mL, but like artificial pleural effusion, about 500 mL is often sufficient.

b. Complications

Bleeding due to direct puncture of the liver is a concern, but only a small portion of the surface is punctured, and hemostasis for bleeding is rarely necessary. On the other hand, CT performed the day after treatment sometimes shows ascites migrating to pleural effusion. It is not an issue if the amount of ascites is about 500 mL, but if ≥ 1000 mL was injected, respiratory distress and decreased SpO_2_ may occur. In such cases, the pleural effusion needs to be drained. It is important to perform CT the day after treatment in order to assess the above.

#### Fusion imaging guidance

A. Background

In the case of ablative therapy performed under US guidance, the keys to successful treatment are stable lesion visualization and accurate targeting on US images. Nevertheless, we often encounter situations where puncture is difficult given the nature of US guidance. Some reasons for targeting mistakes include an indistinct lesion, confusion with coarse regenerative nodules or treatment scars, and visualization range or acoustic window adverse conditions, with identification being difficult and visualization being poor in 2–38.8% of liver cancer cases [14–16].

B. Image fusion system

Various imaging technologies have been invented to overcome the issue of identification difficulties and poor visualization on US. One such technology is image fusion. Image fusion allows us to depict CT or MRI multi-planar reconstruction (MPR) images as pseudo US images while synchronizing the movements of the probe on the US monitor.

**Fig. 7:**
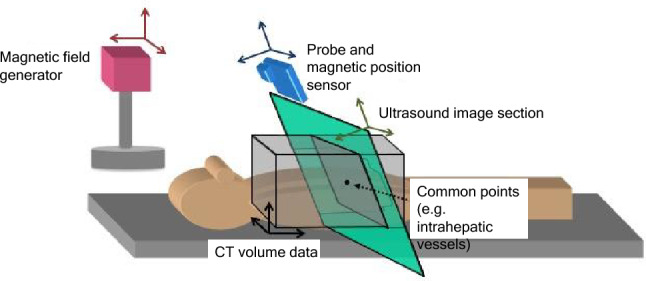
Image fusion system. The position and angle of the probe can be detected in real time in 3D space by means of a position detection system using magnetism. By specifying the shared sites (e.g., intrahepatic vessels) on CT volume data and the US image, an MPR image synchronized with a virtual US image could be demonstrated

It is now possible to capture high-resolution images using multi-detector row CT (MDCT) equipped with multiple detectors, and improvements in computer processing power and advances in computer graphics have greatly improved three-dimensional (3D) imaging technologies. And thanks to magnetic tracking technology, it is now possible to accurately recognize positions in 3D space based on spatial information (relative distance and angle) between a magnetic field generator and a magnetic position sensor. By sharing position information between CT or MRI volume data acquired in advance and US images captured with a probe equipped with a magnetic sensor, it is possible to display B-mode images and approximated MPR images in real time while synchronizing with the movements of the probe (Fig. 7).

The first fusion imaging system was Real-time Virtual Sonography (RVS), which was launched by Hitachi Medico (currently Fujifilm Corporation) in 2003. Before that, US, CT, and MRI were separate independent image information, but the emergence of RVS allowed them to be utilized interactively. Today, the diagnostic units marketed by US manufacturers come equipped with image fusion systems. GE Healthcare has Volume Navigation (V-Nav), Canon Medical Systems has Smart Fusion, Philips has PercuNav, and Siemens has eSie Fusion Imaging.

With respect to image alignment, position adjustment was conventionally initiated based on cross-section synchronization of CT/MRI and US, but a breakthrough was made when image alignment became possible with a single sweep scan based on 3D recognition of the surface structure of the liver or intrahepatic vascular course using Philips' Auto Registration or Canon Medical Systems' PV Detection. In addition, a variety of technologies that support the operator/examiner have appeared, such as an active tracker function that simplifies the synchronization operation and a GPS function that can show the direction and distance to a region of interest designated on the US image.

C. Clinical application as treatment support
A. CT/MR-US fusion imaging

**Fig. 8:**
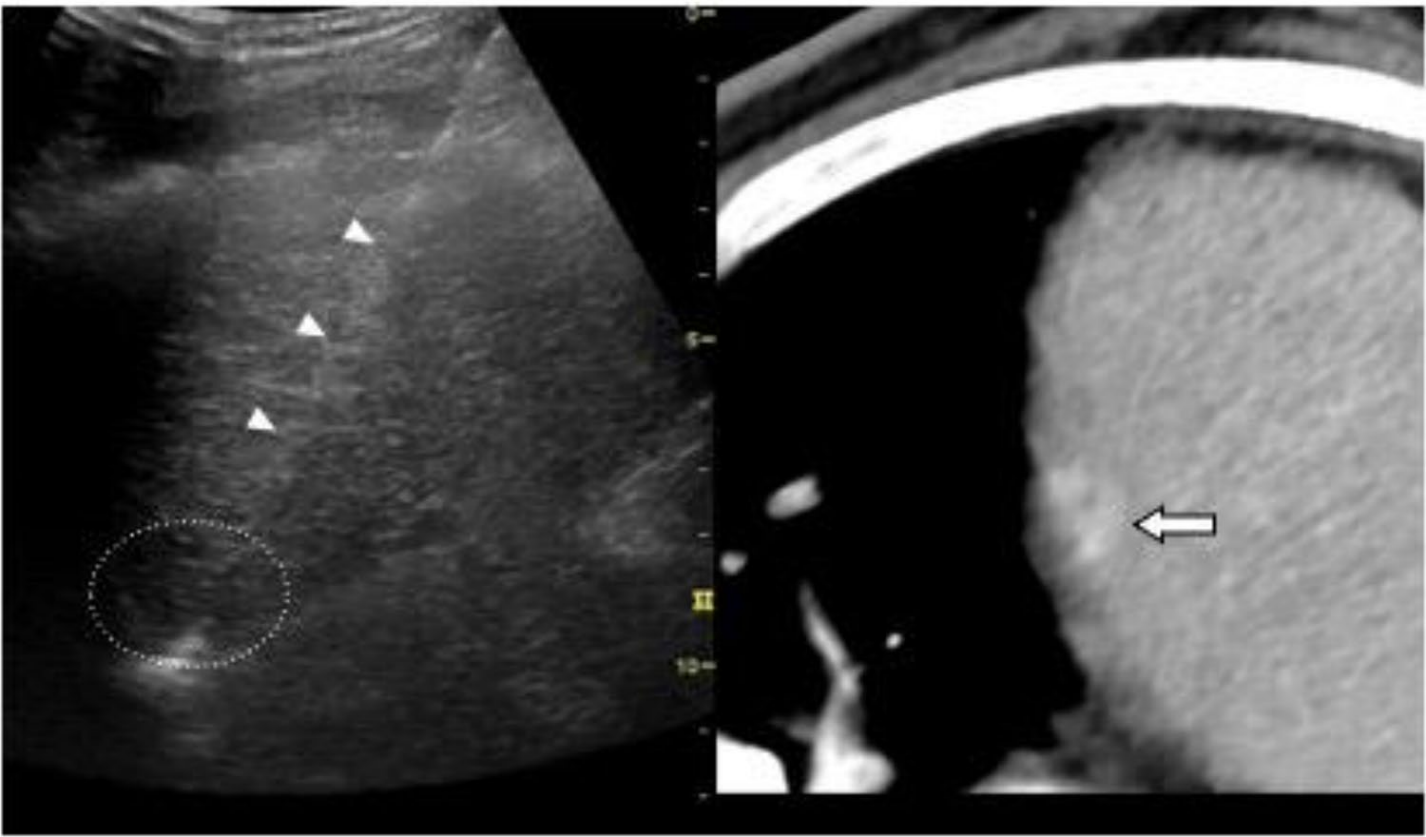
CT-US fusion imaging guidance. A hypoechoic nodule (round dotted line) with an indistinct tumor contour is shown on the US image (left side of screen) so that it corresponds with the densely stained HCC (arrow) on contrast-enhanced CT (right side of screen). This nodule is pierced with the RFA needle (arrowheads)

CT/MR-US fusion imaging, which synchronizes CT/MRI and US, improves visualization and supports the targeting of HCC that is poorly depicted on US. Some specific advantages of CT/MRI-US fusion imaging guidance are that it provides grounds for identifying tumors that are indistinct on B-mode US, facilitates puncturing with the electrode needle at the correct tumor position (Fig. 8), and reduces the number of sessions of local ablative therapy.

The success rates and local recurrence rates for ablation therapy performed under CT/MR-US fusion imaging guidance for HCC indistinct on B-mode US have been reported to be 94.4–100% and 0–8.3%, respectively [17–22].

The following points should be noted during CT/MRI-US fusion imaging guidance.

(i) Misregistration

The state of the liver is not static; the liver can twist and change its shape. During image synchronization, CT/MRI and US images do not completely match in many instances, so one should assume that there will always be a certain degree of misregistration. In order to minimize misalignment of CT/MRI and US images, image adjustment needs to be carefully repeated, using an intrahepatic vessel as close to the tumor as possible as a guidepost.

(ii) Timing of image synchronization

When the patient repeatedly breathes with maximum inspiration and maximum exhalation, respiration depth will not be constant due to “breathing fatigue,” making image synchronization challenging. There will be little breathing error when image synchronization is carried out at expiratory standstill during natural breathing.

b. New applications of fusion imaging
(i) 3D Sim-navigator and E-field

**Fig. 9:**
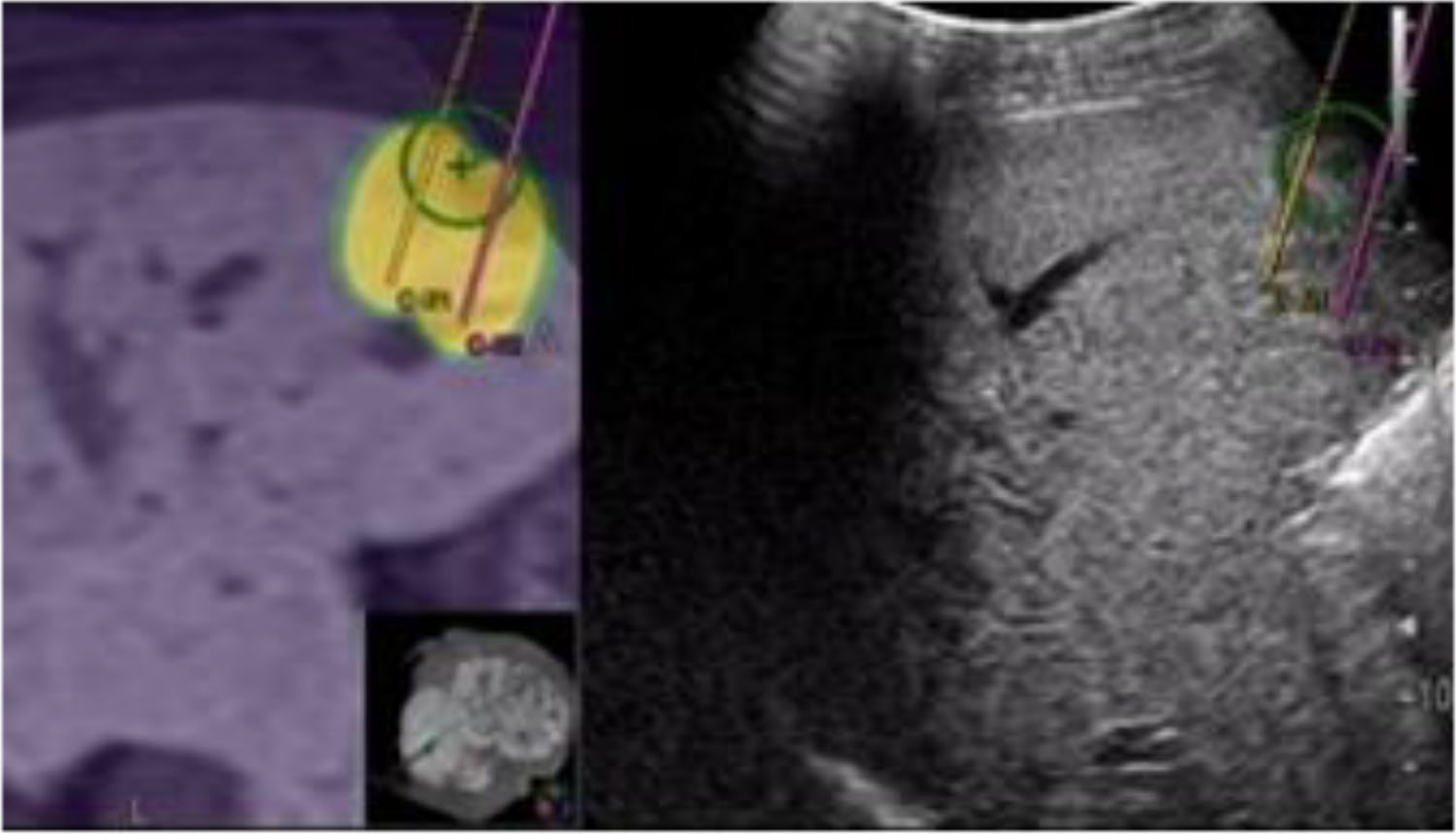
Simulation using 3D Sim-Navigator and E-field. The expected ablation area (yellow) when the RFA needle is inserted along two virtual puncture lines (yellow and purple) is displayed. The simulation image can also be utilized for treatment navigation as a reference screen

In the case of bipolar RFA using Celon Power, multiple electrode needles need to be inserted in a way that surrounds the tumor, but a sufficient ablation area cannot be achieved if the distance between the needles is too long. Because the distance between the needles must be suitable, bipolar RFA is a difficult procedure to perform. 3D Sim-Navigator is comprised of puncture simulation, in which multiple virtual puncture lines are drawn on top of CT/MRI volume data, and puncture navigation, during which the virtual puncture lines can be referenced. Thanks to the good compatibility between the highly robust Celon Power applicator and 3D Sim-Navigator, they are highly useful as treatment support for bipolar RFA [23]. In addition, E-field can be used to illustrate the expected extent of ablation in RFA treatment, and it can be utilized to simulate ablation therapy according to virtual puncture lines (Fig. 9).

(ii) US-US fusion imaging and US-US overlay fusion

**Fig. 10:**
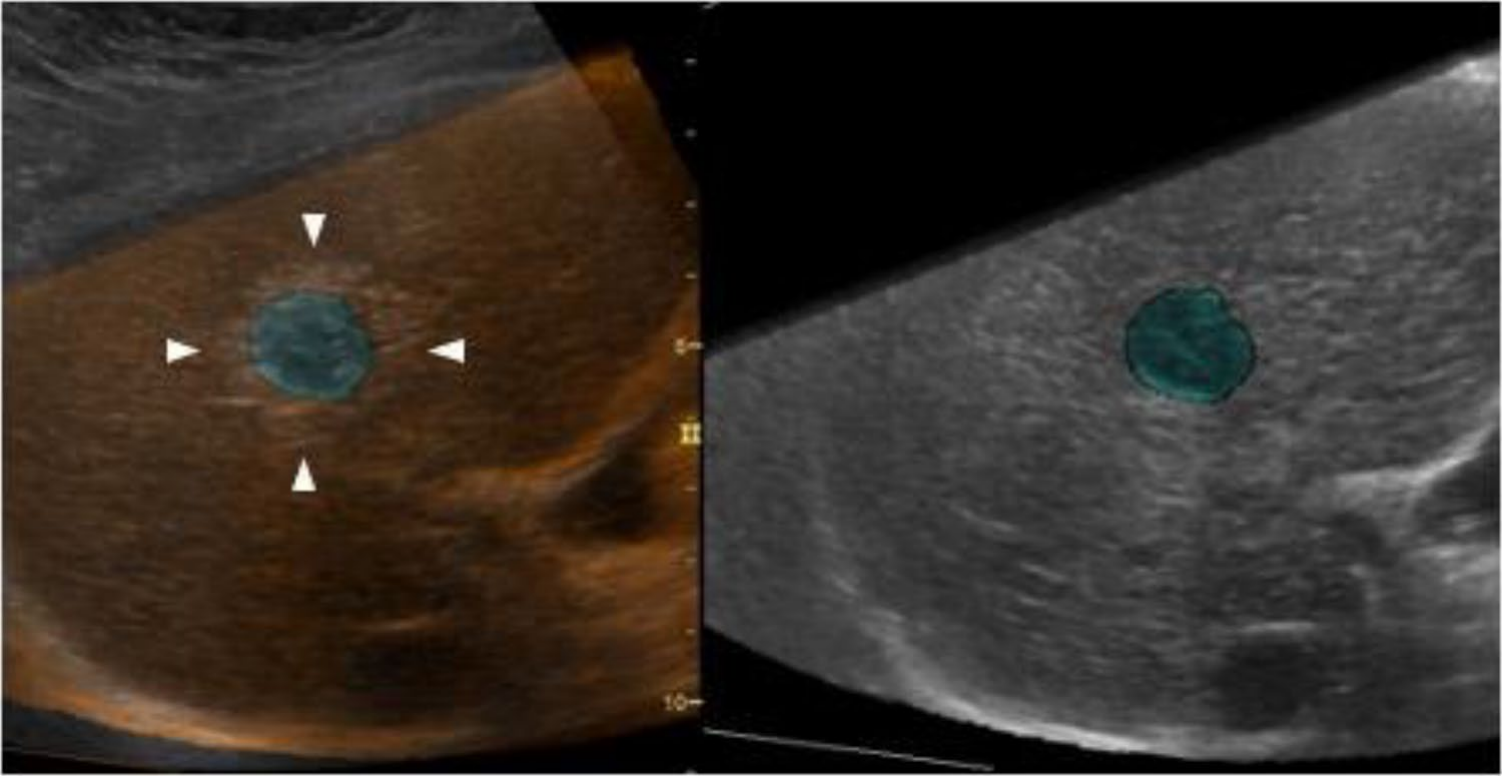
US-US overlay fusion. The right side of the screen is the baseline US image, where the HCC nodule is indicated in green. On the left side of the screen, the baseline US image is projected on a US image taken immediately after treatment using the overlay function (image overlay). A region differentiating the ablative hyperechoic area and the green tumor image can be detected as the margin

During ablation therapy, it is difficult to evaluate the ablative margin in detail during treatment because the tumor is hidden under a hyperechoic area that appears in association with ablation. However, there is a real risk of missing areas with an insufficient margin as the decision to end ablation therapy is subjectively made based on US images. On the other hand, it is now possible to overlay US images captured before and after ablation for comparison thanks to advances in imaging technology [24–28]. In a word, side-by-side comparison (US-US fusion imaging) and overlaying (US-US overlay fusion) are possible by synchronizing MPR images from US volume data captured immediately before ablation and US images taken immediately after ablation, allowing for identification and visualization of margins (Fig. 10). RFA treatment under US-US overlay fusion guidance improved the safety margin achievement rate (89.3% vs. 47.0%, P < 0. 01) and also curbed local recurrence (2-year local recurrence rate: 0.8% vs 6.0%, P = 0.022) [29]. Given that ensuring the safety margin was relatively easy even in the case of tumors exceeding 2 cm in diameter, in particular, the utility of using US-US fusion imaging/overlay fusion guidance may be greater when treating HCC on the large side.

“Visualization of the ablative margin” is synonymous with “(early) assessment of treatment response”. CT exposure and contrast agent-induced renal impairment are concerns in HCC patients with long-term duration of disease as they undergo many imaging tests during their lifetimes. For that reason, it is preferable to refrain from performing contrast-enhanced tests, but the fact of the matter is that contrast-enhanced CT/MRI is performed within a week after ablative therapy for early assessment of treatment response at many institutions in Japan. Although the percentage of cases in which images can be precisely aligned using US-US overlay fusion is limited, margin evaluation (monitoring) using US-US overlay fusion can be a substitute for contrast-enhanced CT/MRI in the early assessment of treatment response, and can contribute to reducing radiation exposure and lowering the risk of renal impairment [30, 31].

#### Contrast-enhanced US

Sonazoid (GE Healthcare Pharma, Tokyo, Japan), the only contrast agent that is covered by health insurance in Japan, is an agent containing perflubutane covered in a phospholipid monolayer shell. It was first used for hepatic mass lesions in January 2007 in Japan. In addition to Japan, it is available in South Korea, Taiwan, Norway, China, and Singapore. The perflubutane bubbles are 2–3 μm in size, which is smaller than red blood cells, and they are metabolized in the lungs and expelled from the body through respiration; therefore, Sonazoid can be used in patients with renal impairment. It needs to be carefully administered in patients with an egg allergy as it uses hydrogenated egg phosphatidylserine sodium as an excipient. The only contraindication in the package insert is "Patients with a past history of hypersensitivity to the constituents of this product," but the frequency of allergic reactions is presumed to be very low. It can also be used in patients who are allergic to contrast-enhanced CT/MRI and patients with asthma.

The recommended dose in the package insert is “Intravenous administration of 0.015 mL/kg as a suspension once in adults”. The dose in a patient weighing 60 kg would be 0.9 mL, for example. This dose is based on the results of a clinical study (using US systems from multiple manufacturers) that was begun in 2002, but the performance of US systems has steadily improved over the 20 years since then; therefore, sufficient contrast can be achieved with a dose smaller than the recommended dose. As a side note, 0.5 mL per body is administered at many institutions in Japan [32–34]. When the examination is performed by a single operator, sufficient contrast can also be achieved by fully opening the IV for a while after injecting Sonazoid from the three-way stopcock.

## Ablation procedure

### Specific procedure

**Fig. 11:**
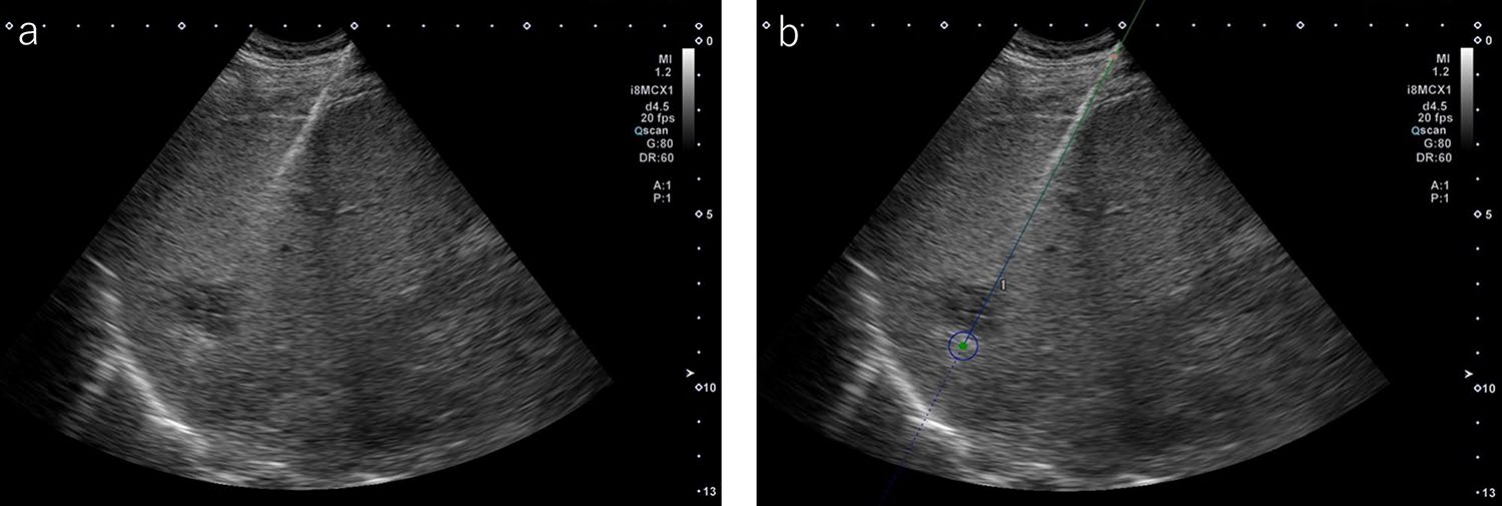
**a** MWA (Emprint ablation system) was performed for HCC in S6, but the tip of the antenna is indistinct. **b** The antenna tip is clearly displayed when a needle navigation system (Cannon Medical Systems) that can display the needle tip is used

Perform ablative therapy as planned during planning. First, local anesthesia is administered under US guidance, but the patient may complain of pain when the liver is punctured with the needle if anesthesia at the liver surface is insufficient. It is important to administer sufficient anesthesia not only to the skin and subcutaneous tissue but also to the liver surface. If a trocar is used, insertion of the needle into the liver will be easier when the trocar is inserted as far as the subcutaneous tissue before puncture with the applicator. This will also reduce the risk of intercostal arterial injury and should be used by beginners. In the case of treatment of a deep-seated lesion, however, the needle may not reach the target lesion due to the long trocar if a trocar is used. In such cases, a workaround will be needed, such as using a longer needle or not using a trocar. After inserting the needle in the liver, advance the needle to the deep area as slowly as possible. Doing so can minimize injury to the hepatic arteries, portal vein, and nerves in Glisson's capsule. If the needle deviates from the planned puncture line, remove the needle up to near the liver surface and puncture again after changing the direction of the needle tip. In the unlikely event that you lose sight of the tip, do not advance the needle any further; pull the needle out to a depth at which you can see the tip of the needle or use a system to display the needle tip on the US screen such as needle navigation (Fig. 11).

### Ablation (RFA) procedure with margins in mind

(i) Importance of RFA needle puncture route and confirmation of placement site

**Fig. 12:**
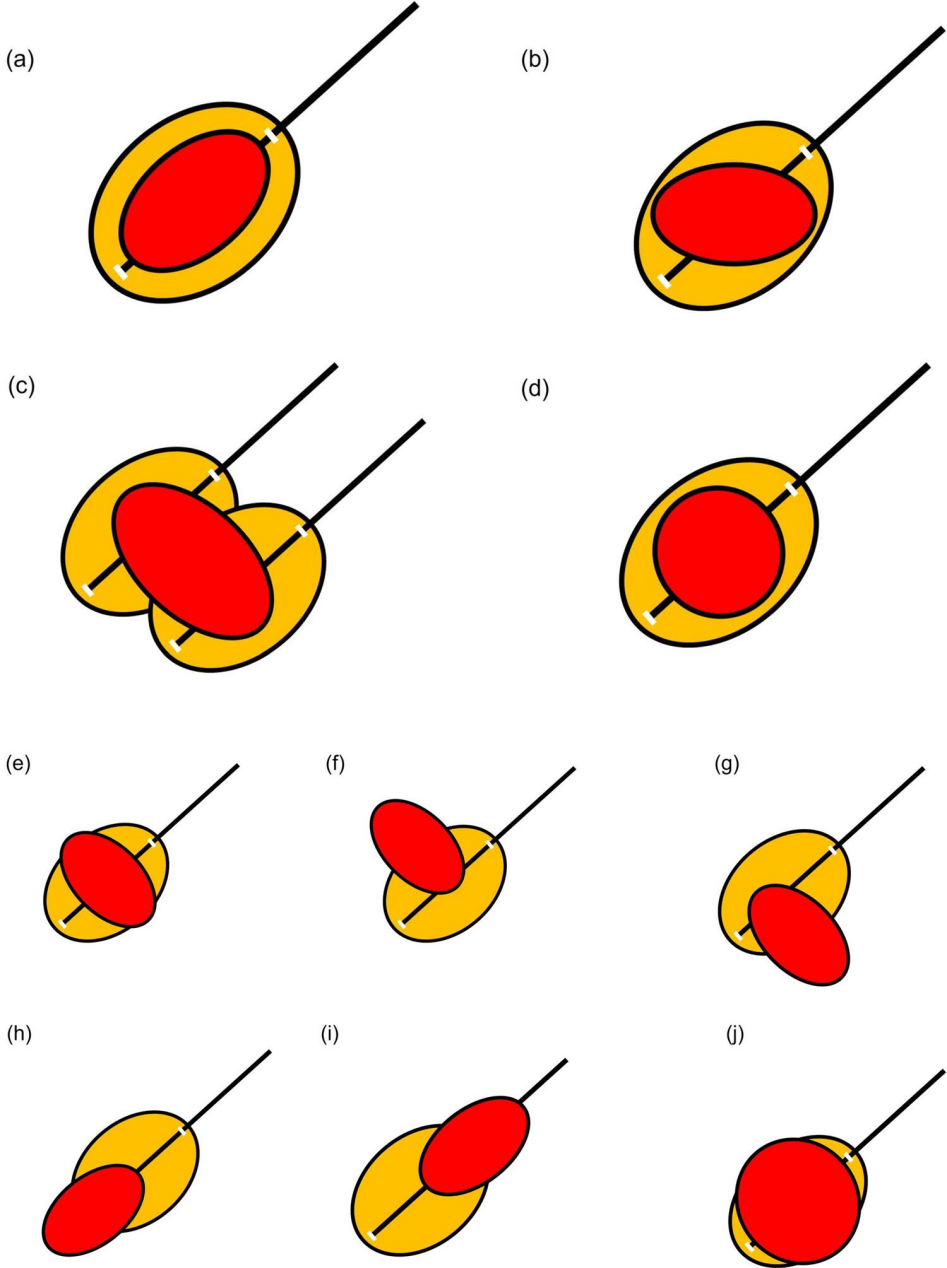
Relationship between placement position of RFA puncture needle and extent of ablation. **a** In the case of an oval lesion, ablation with a sufficient margin can be achieved if it is punctured in the long axis direction. **b** In practice, it is not always easy to puncture an oval lesion with the long axis perfectly aligned. However, ablation with a sufficient margin can be achieved by inserting the needle along the long axis of the lesion, as shown in the figure. In some cases, one should consider setting the puncture line by inverting the probe left or right when puncturing the lesion with the needle. **c** If it is only possible to set the puncture line to be perpendicular to the long axis direction of the lesion, radiofrequency ablation is performed with multiple punctures as shown in the figure. **d** In the case of a lesion with a shape that is nearly a perfect circle, the margin should be made slightly larger, performing ablation in an oval manner. **e** If the needle is punctured so that it is perpendicular to the long axis of the oval lesion, the concern is that there is insufficient margin in the long axis of the lesion. **f** If the puncture is made far from the center of the lesion, the lesion may remain on the opposite side of the puncture site. **g** As opposed to the puncture in (**f**), it will be difficult to evaluate the site with suspected residual tumor due to post-ablation gas in the case of the puncture in (**g**). Therefore, moving the needle tip in the direction in (**g**) must be avoided during puncture. **h** Even if the puncture can be successfully performed in the longitudinal direction, there is a possibility of residual tumor deep in the lesion when ablation is performed at the front side of the lesion due to not being able to recognize the needle tip. Visualization of the deep part on US becomes difficult as gas appears with ablation of the superficial part, making evaluation of the part with suspected residual tumor challenging. Performing additional ablation of the deep part must be avoided as evaluation of the part with residual tumor and visualization of the needle tip both pose a high level of difficulty. **i** Ablation of the deep part of the lesion can leave residual tumor in the superficial part of the lesion. There is gas caused by ablation in the deep part, but the superficial part can be visualized, making it relatively easy to perform additional ablation. Therefore, performing a slightly deeper puncture makes it easier to evaluate the part with residual tumor and perform additional ablation. **j** In the case of a lesion that is a perfect circle, proceeding with treatment without performing ablation in an oval manner can leave residual tumor in the radial margin. In this case, it will be difficult to evaluate the margin on the deep side. Therefore, if the puncture line will likely be like that in (**e**), it will be important to envision performing ablation of the entire lesion by performing a puncture like that in (**f**), evaluating the presence or absence of residual tumor in the superficial part of the lesion with contrast-enhanced US, and then performing a puncture like that in (**c**). If the puncture line is expected to be like that in (**j**), it will be important to either make the longitudinal margin bigger or envision ensuring the margin by performing two punctures, as in (**c**). From the standpoint of increased difficulty of post-ablation evaluation, the puncture lines that need to be avoided most are those in (**g**) and (**h**), while the puncture lines in (**f**) and (**i**) can be corrected given that post-ablation evaluation is possible. However, the needle tip reaches the deep part in both (**f**) and (**i**); therefore, one must be attentive to complications such as vascular injury. Red indicates the tumor (lesion), and orange represents the extent of ablation

**Fig. 13:**
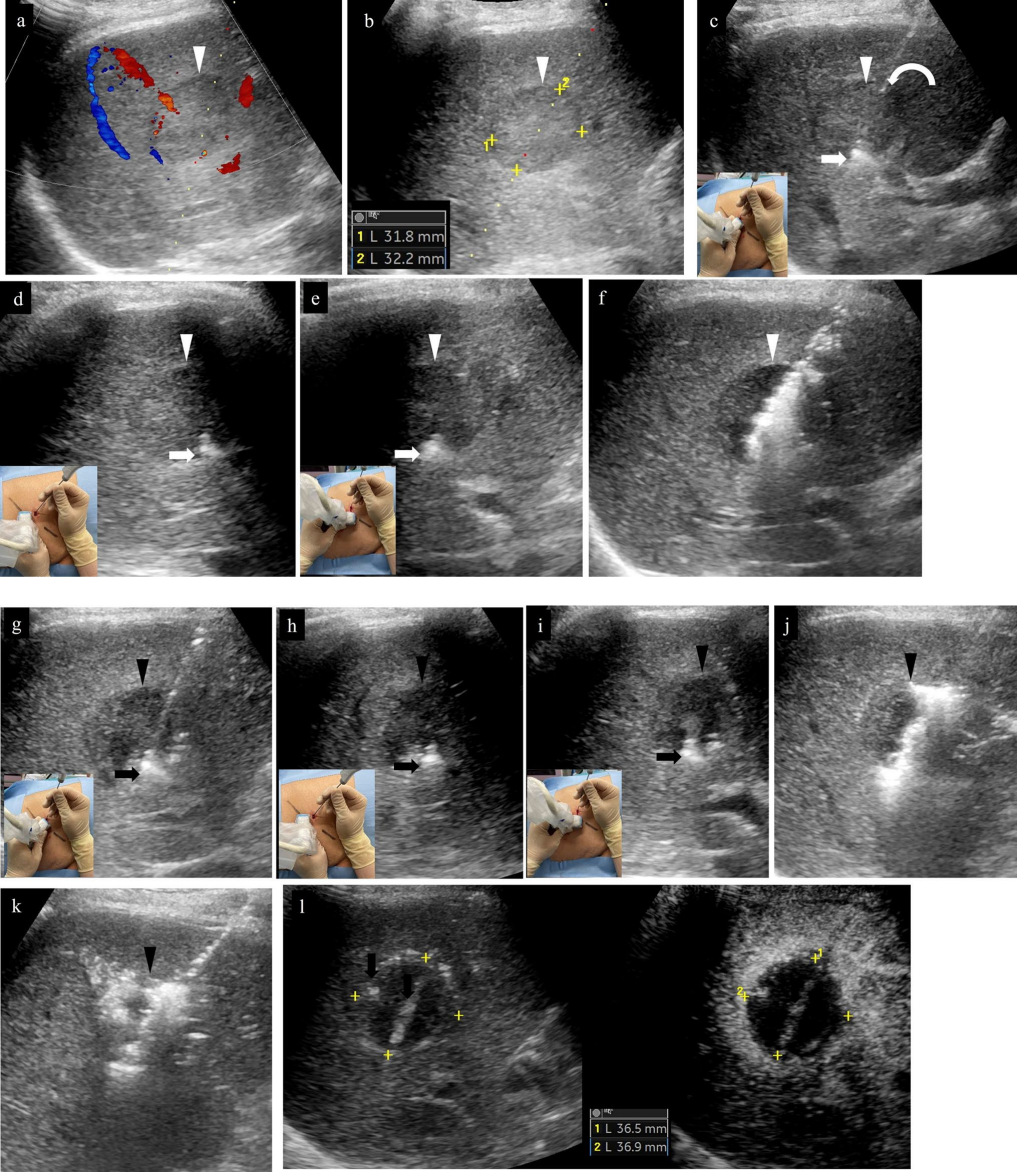
RFA for hepatocellular carcinoma (right hepatic lobe anterior segment, tumor diameter 32 mm) and assessment of response immediately after treatment. **a** Check for the presence or absence of vessels on the puncture line via color Doppler US as a pre-treatment simulation. **b** Since the maximum diameter of the tumor is 32 mm and the longest diameter on the puncture line is about 32 mm, 3 cm was selected as the extent of ablation with the puncture needle. **c** An image of the puncture taken with intercostal scanning is shown. The tip (tip of current-carrying part, arrow) of the puncture needle and the end of the current-carrying part (arrowhead) are shown in the figure. The needle is located slightly to the right of center of the tumor when facing the screen. **d** The needle tip is seen on the right side of the tumor when the probe is placed horizontally to the patient's body and the tumor is observed in the horizontal direction. A portion of the right edge of the tumor cannot be seen due to being obscured by the ribs. **e** When the probe is rotated 90 degrees clockwise from the above-mentioned placement to observe the tumor in the vertical direction, it can be understood that the caudal side of the lesion will be primarily ablated given that the needle will be inserted in the tumor from the caudal side of the tumor and the needle tip is seen in the caudal side of the tumor. From the above, it can be understood three-dimensionally that the right caudal side of the target lesion will be ablated. **f** Gas caused by steam is observed at the center of the tumor during RFA treatment. **g** Second puncture. The second needle was inserted further to the right than the first needle. **h** In the case of intercostal scanning, the needle tip is seen on the right side of the tumor and more caudally than the first puncture when observing the tumor horizontally while scanning horizontally since the right side is punctured. A portion of the right edge of the tumor cannot be seen due to being obscured by the ribs. **i** The needle is inserted from the caudal side of the tumor and the needle tip is seen caudal to the center of the tumor when the tumor is observed in the vertical direction. The needle cannot be confirmed in the same cross section after the first needle as the puncture is made to the right of the first puncture site. **j** Steam gas is observed primarily on the right side of the tumor during RFA treatment. **k** The third puncture was made at the cranial midline of the tumor, and the fourth puncture was made slightly cranially and to the left side of the tumor. Gas caused by steam is observed in the entire tumor during the third ablation. **l** Treatment was ended after confirming the absence of contrast enhancement in an area larger than the original tumor in the arterial phase of low mechanical index contrast imaging performed to assess treatment response immediately after RFA. Steam gas is hyperechoic as it is immediately after treatment, and the image is slightly hyperechoic for that reason during contrast enhancement. Strict follow-up observation is necessary as the margin is less than 5 mm. Note that recurrence is not found 6 months after RFA. Arrowheads indicate the tumor border

**Fig. 14:**
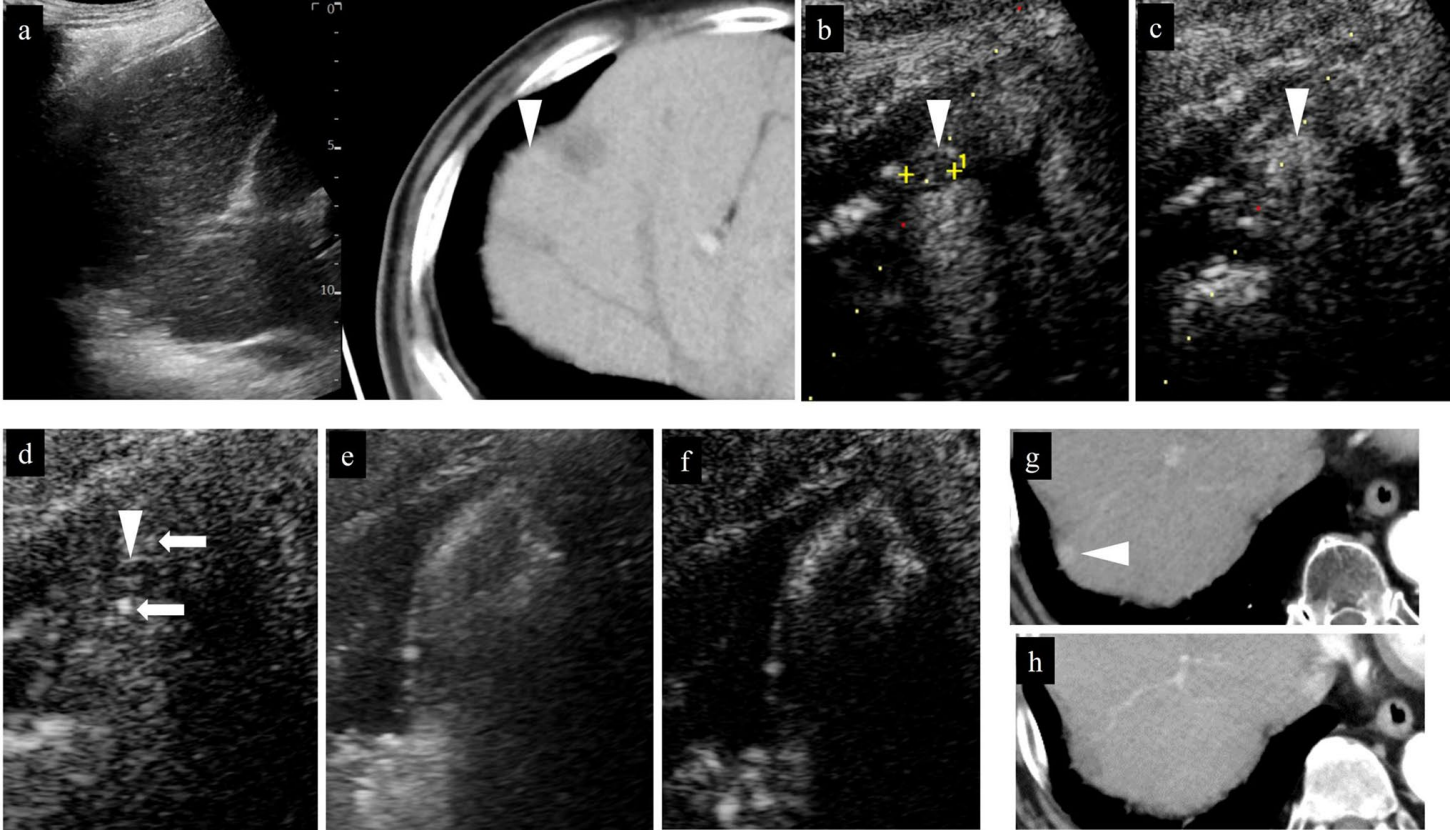
Right hepatic lobe posterior segment, recurrence of hepatocellular carcinoma (tumor diameter 10 mm) in vicinity of RFA. **a** On a fusion image of B-mode US and contrast-enhanced CT, a small site of recurrence in the vicinity of the RFA treatment site is seen in the arterial phase of contrast-enhanced CT (arrowhead), but the lesion itself cannot be detected due to being obscured by the lung on B-mode US. **b** The lesion was depicted as hypoechoic when it was observed in the post-vascular phase of low mechanical index contrast imaging after injection of artificial pleural effusion to ensure the visual field. **c** The same site was densely stained after a second intravenous injection of Sonazoid and recognized as the target hepatocellular carcinoma. **d** The hypoechoic lesion was targeted for puncture in the post-vascular phase of contrast-enhanced US. The markers for the tip and distal side of the puncture needle are depicted (arrows). **e** Steam gas was observed after performing RFA treatment at the same site. **f** Contrast enhancement is not seen at the RFA site on low mechanical index contrast imaging performed immediately after RFA. Based on a comparison of the arterial phase of contrast-enhanced CT before treatment (**g**) and the arterial phase of contrast-enhanced CT 1 month after treatment (**h**), it was determined that sufficient ablation was achieved. The arrowhead indicates the tumor border

**Fig. 15:**
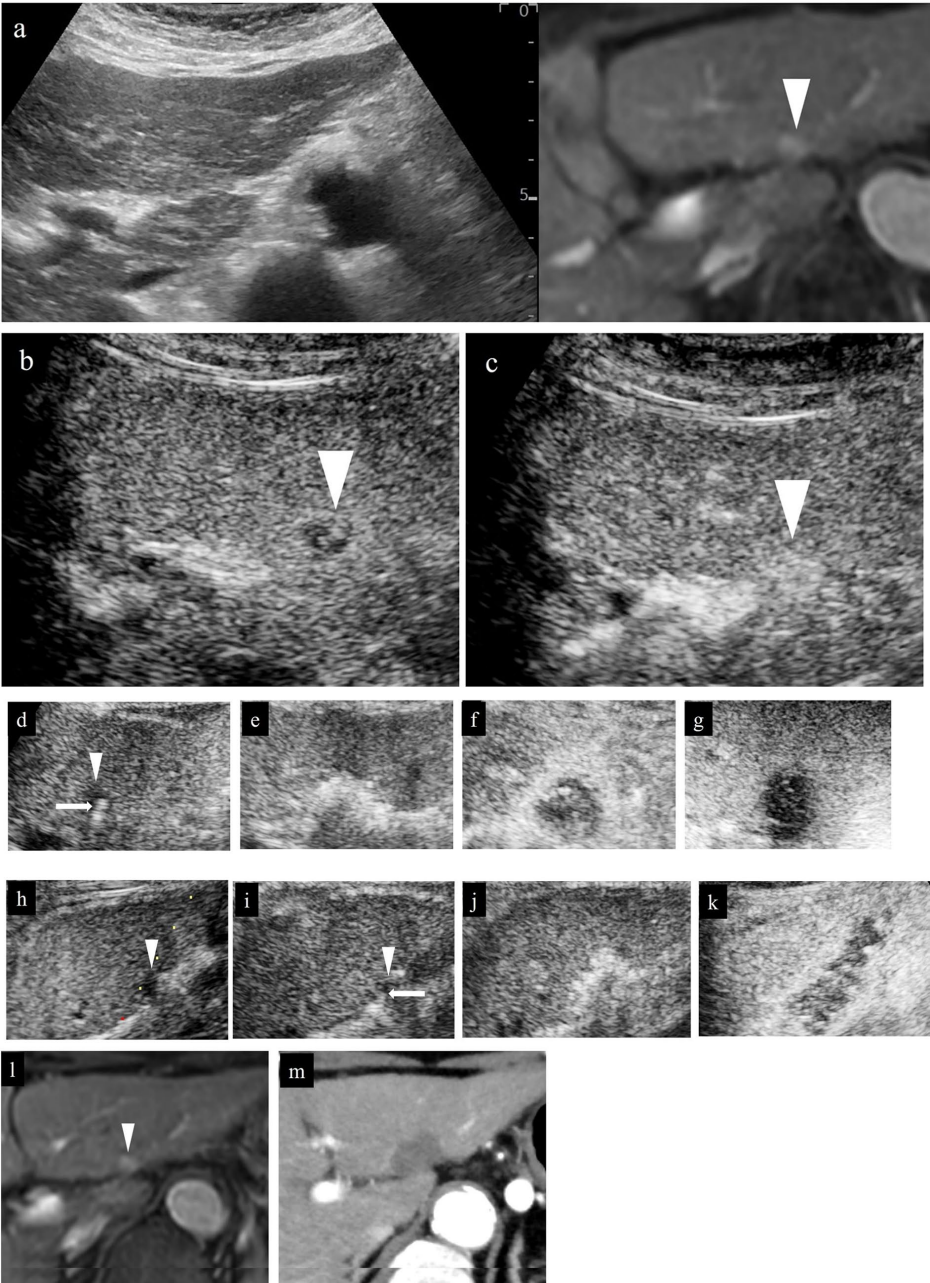
Hepatocellular carcinoma (left lobe lateral segment, tumor diameter 10 mm) that was difficult to detect on B-mode US. **a** On a fusion image of B-mode US and the arterial phase of EOB-MRI, the tumor cannot be detected on B-mode US at the site that corresponds to the site that is densely stained in the arterial phase of EOB-MRI. **b** The lesion was depicted as hypoechoic in the post-vascular phase of low mechanical index contrast imaging. **c** The same site was recognized as the target lesion after being densely stained with a second injection of Sonazoid. **d** The RFA needle (extent of ablation 15 mm) was inserted at the hypoechoic site in the post-vascular phase. Images **d**–**g** show transverse scanning after puncture. The puncture needle (arrow) is seen inside the tumor. **e** With respect to the target lesion, steam gas is found in an area more widespread than the hypoechoic site in the post-vascular phase. **f** The same site is not densely stained in the arterial phase of low mechanical index contrast imaging performed immediately after RFA, but the area around RFA was enhanced due to inflammation, etc. **g** On high mechanical index contrast imaging performed immediately after that, a wider area is observed as an unstained area as it is less affected by B-mode. Images **h**–**k** are images of longitudinal scanning before and after puncture. It is hypoechoic (**h**) in the post-vascular phase, and the puncture needle (arrow) is seen inside the tumor after puncture. **j** During ablation, widespread steam gas is seen in the target lesion. **k** The same site is not densely stained in the arterial phase on low mechanical index contrast imaging performed after RFA. Route ablation was performed when pulling out the needle as the portal vein was present on the puncture route. Sonazoid contrast enhancement is not found on the body surface side. **l, m** The arterial phase of EOB-MRI before RFA (**l**) and the arterial phase of contrast-enhanced CT 1 month after RFA (**m**) indicate that the tumor was properly treated. Arrowheads indicate the tumor border

**Fig. 16:**
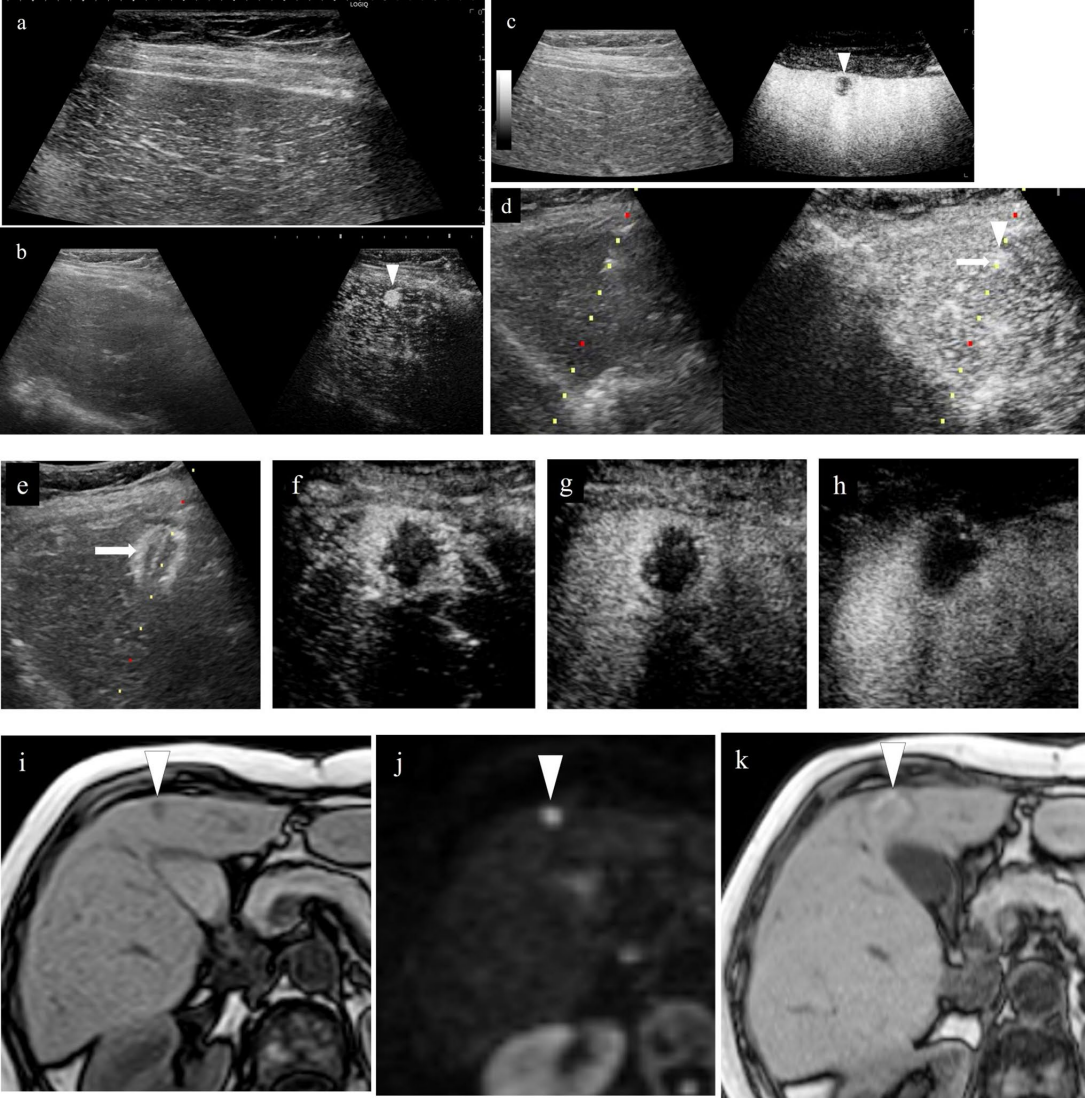
Hepatocellular carcinoma (left lobe medial segment, tumor diameter 10 mm) that was difficult to detect on B-mode US. **a** The lesion could not be detected on B-mode US with a linear probe. **b** Dense staining was seen in the arterial phase on low mechanical index contrast imaging. **c** It was depicted as hypoechoic in the post-vascular phase. **d** Puncture was performed in the arterial phase on low mechanical index contrast imaging on the day of RFA. The needle is seen in the densely staining part (arrow). **e** An insertion mark is seen on B-mode US after gas caused by ablation had disappeared. **f** An unstained area larger than the original lesion was seen in the arterial phase on low mechanical index contrast imaging performed after RFA. **g** No clear contrast findings are seen in the punctured area in the portal venous phase either. **h** Contrast enhancement is not seen up to the liver surface at the punctured area when observing the area while destroying bubbles in the portal venous phase on high mechanical index contrast imaging. With respect to the site stained by inflammation adjacent to the ablation zone in the arterial phase, high mechanical index contrast imaging was more effective for evaluation of the extent of ablation than low mechanical index contrast imaging as it is less affected by background B-mode US. In this case, evaluation was performed with simple MRI before RFA as a contrast agent could not be used with MRI due to the patient's chronic renal failure. In the medial segment of the left lobe, small hypointensity (**i**) was seen on the T1-weighted image, and small hyperintensity (**j**) was observed on the diffusion-weighted image. A change in the signal was seen in an area sufficiently larger than that of the original tumor on the T1-weighted image of simple MRI performed 1 month after RFA (**k**). This is thought to reflect the extent of necrosis. Arrowheads indicate the tumor border

Where one places the needle in the tumor at the puncture stage is important when assessing treatment response to RFA. It is generally rare for ablation effect to occur in a perfect circle in RFA; ablation effect usually occurs elliptically along the long axis of the puncture needle (Fig. 12). This may be due to a cooling effect from vessels distributed throughout the liver. Therefore, it is important to confirm the shape of the lesion before starting RFA and perform the puncture after considering which puncture direction will produce a sufficient ablation area. In other words, in addition to searching for a puncture line that will avoid blood vessels using US immediately before performing RFA, one needs to confirm the shape of the lesion and look for a puncture line that will achieve ablation effect with a sufficient margin. In addition, one needs to select an electrode that will yield an ablation area large enough for the target extent of ablation. When a deployable needle electrode is used, it should be noted that ablation occurs over a wide area in the short-axis direction. When an electrode with a wide ablation area on the long axis is selected, the ablation area on the short axis will also generally be wider as a result. In addition, in order to accurately evaluate the position of the needle relative to the lesion during puncture, one should consider confirming the lesion using not only intercostal scanning but also longitudinal scanning (patient head–tail direction) and transverse scanning (patient CT axial cross section) (Fig. 13). We often experience situations where we think we have placed the electrode in the center of the lesion only to discover that it actually deviates from the center when confirmed on transverse scan [35]. It is not difficult to identify the position of the needle when the tumor is satisfactorily depicted on B-mode US (Fig. 13), but the position should be evaluated using contrast-enhanced US when visualization is not adequate (Figs. 14, 15, 16). If the needle has pierced the lesion, sufficient ablation can at least be achieved in the longitudinal direction.

(ii) Relationship between placement position of RFA puncture needle and extent of ablation

Confirm the morphology of the lesion with not only prior US but also CT or MRI, ascertain the position that represents the largest side and long axis of the lesion, and aim to perform ablation with the electrode placed at the position that represents the long axis. To that end, one should strive to never lose sight of the tip of the needle on US during puncture and ablation. If the tip of the needle cannot be visualized during RFA, the risk of complications such as injury to blood vessels and other organs increases, and when the needle moves due to respiration, the planned ablation cannot be achieved and assessment of treatment response will be challenging. When steam gas is generated during ablation, the US beam cannot reach deep areas and it becomes difficult to see the needle tip, but it is important to always keep sight of the needle tip until then. In addition to visualization of the needle tip, if there is a graduation that indicates distance marked on the surface of the needle axis, confirm the position on the patient's skin surface before starting ablation and check whether the insertion depth of the needle has changed. If ablation can be performed with the electrode placed at the position that represents the largest side and long axis of the tumor, it will be relatively easy to ensure the longitudinal margin (Fig. 12). In the case of RFA, adequate treatment is generally regarded as achievement of thermocoagulative necrosis that includes the target lesion (HCC) and a margin ≥ 5 mm including the surrounding non-tumor area [36].

(iii) Importance of ensuring radial margin

Since ablation extends 2–3 mm beyond the distal end and proximal end of the electrode, respectively, ablation with a margin of 5 mm, respectively, can be achieved by adjusting the electrode with that fact in mind. The challenge here is ensuring the radial margin relative to the electrode. If it is assumed that ablation can be achieved in a perfect circle, ensuring the radial margin would be easy, but it is actually difficult. In other words, the most important factor that determines whether an adequate ablation area is achieved is whether the radial margin can be ensured (Fig. 12).

(iv) Puncture method for lesions with different shapes

This is explained using a schematic (Fig. 12). Before initiation of ablation, save a still US image that indicates the position of the needle and the position of the tumor, and measure the distance from the distal end to the proximal end of the electrode. Then, measure the maximum length from the longitudinal to vertical direction beforehand, and after ablation, confirm with contrast-enhanced US whether a margin at least 5 mm beyond the maximum length was achieved (Fig. 12a). As a practical matter, however, there are many lesions that are difficult to puncture longitudinally depending on the position of the lesion. The puncture should be made as longitudinally as possible (Fig. 12b), but if it is difficult to do so, one should try to ensure the margin by means of multiple punctures, etc. (Fig. 12c). If the shape of the lesion is nearly a perfect circle, it is often possible to ensure the radial margin by making the longitudinal margin a little bigger (Fig. 12d).

(v) Impact of puncture needle movement due to respiratory fluctuations and impact of steam gas caused by ablation

The position of the electrode may move due to respiratory fluctuations during ablation. In that case, not only will the extent of longitudinal ablation differ from what is expected but the radial margin will also be insufficient. In addition, the US beam will be less likely to reach deep areas when steam gas occurs due to ablation. This needs to be kept in mind when performing ablation. In the case of a tumor that requires two punctures, the deep side should be punctured and ablated first, and then the shallow side should be punctured after confirming whether there is any residual tumor on the shallow side by evaluating the ablation area using contrast-enhanced US. When the shallow side is ablated first, it becomes difficult to visualize the lesion on the deep side due to steam gas. The lesion can eventually be visualized as the steam gas dissipates over time, but visualization will be more challenging than before ablation; therefore, the next ablation will be easier if one leaves the shallow side until after the deep side. Therefore, rather than use the puncture line shown in Fig. 12e, one should first perform the puncture shown in Fig. 12f, and then perform ablation of the entire lesion by performing the puncture shown in Fig. 12g. When the puncture line is expected to be like the one in Fig. 12j, it is important to either make the longitudinal margin bigger or ensure the margin by performing two punctures, as in Fig. 12c. From the standpoint of increased difficulty of post-ablation evaluation, the initial puncture lines that need to be avoided most are those in Fig. 12g and h; the puncture lines in Fig. 12f and i are often amenable to correction given that post-ablation evaluation is possible. On the other hand, the needle tip reaches the deep area in the case of the punctures shown in Fig. 12f and i; therefore, one must be attentive to complications such as vascular injury.

(vi) Importance of selection of intercostal space for puncture and concomitant artificial pleural effusion

Besides which puncture line to take, selection of the intercostal space is also very important. First, it is important to take a puncture line that is vertical to the patient's body to eliminate the impact of respiratory fluctuations. To that end, select an intercostal space that is as cranial as possible. When the puncture is performed while scanning with the probe in a manner that looks up at the lesion from between the ribs, the position of the electrode will not remain fixed due to being heavily impacted by respiratory fluctuations, which will make it more likely that the ablation area will be insufficient. Moreover, there is a risk of injuring intercostal arteries during the puncture and causing bleeding complications. Inexperienced operators, in particular, tend to perform the puncture at the first intercostal space through which they visualize the target lesion, but we recommend trying to visualize the target through one intercostal space closer to the head before performing the puncture.

On the other hand, it can be challenging to recognize the lesion due to being obscured by the lungs when the puncture is performed in a direction that looks down at the lesion through a cranial intercostal space. If is often impossible to recognize a lesion right below the diaphragm due to being obscured by the lungs unless scanning with the probe is done in a manner that looks up between ribs. In such cases, using artificial pleural effusion will eliminate the influence of lung gas, making visualization from between a cranial intercostal space possible (Fig. 14).

(vii) Concept of ablative margins

In the case of primary HCC, a sufficient margin of ≥ 5 mm is considered to be necessary, but in cases with repeated recurrence, this rule is not always strictly followed, in part out of consideration for preserving liver function. It is often difficult to predict the extent of ablation due to the cooling effect from blood vessels around the lesion and other factors. In cases where the ablation area is not as large radially as expected, ablation should be added as necessary. When doing so, it is important to keep in mind that it will be difficult to visualize deep areas due to steam gas, potentially resulting in residual tumor.

## Ablation under computed tomography (CT) guidance and endoscopic ultrasonography (EUS) guidance

### CT guidance (including CT assistance)

CT guidance is an option for tumors that are difficult to visualize with US. The procedure for CT guidance is as follows. Capture a CT image after affixing a marker to the body surface of the patient, calculate the puncture angle at the insertion site and the distance based on the positional relationship between the tumor and the marker, and penetrate the tumor with the applicator under CT guidance or while capturing CT images (CT assistance) as needed. Note that performing transcatheter arterial chemoembolization prior to ablation will facilitate lesion identification as a result of accumulation of lipiodol in the tumor, which will aid puncture and assessment of response [37].

### EUS guidance

Ablation of hepatic tumors in S1 (caudate lobe) is challenging via a percutaneous approach as they are located far below the body surface, but they can sometimes be easier to approach due to a shorter distance via a gastrointestinal route. Since the movement of the puncture needle can be monitored on the US screen with convex EUS, transgastrointestinal intervention under EUS guidance is possible. Such procedures are collectively referred to as interventional EUS, but EUS-guided ethanol injection therapy is also performed for S1 (caudate lobe) hepatic tumors [38].

## Assessment of treatment response

### Assessment of response to HCC ablation

Contrast-enhanced CT is generally used for evaluation. Perform contrast-enhanced CT the day after treatment to not only evaluate the ablative effect but also investigate the presence or absence of complications. On the other hand, there are various issues associated with contrast-enhanced CT, such as increased exposure to radiation, occasional difficulty performing evaluation due to treatment-emergent arterioportal shunt, and decreased revenue due to the cost of imaging during hospitalization. In addition, not only can the patient not be billed for costs related to additional ablation for residual tumor including a second needle but revenue and expenditures shift in the negative direction as a result of a decrease in national health insurance points due to prolongation of hospitalization. For those reasons, some institutions use contrast-enhanced US during treatment to assess treatment response and determine whether or not to end treatment, and then perform contrast-enhanced CT 1 month after treatment.

### Assessment of response during RFA using contrast-enhanced US and associated issues

**Fig. 17:**
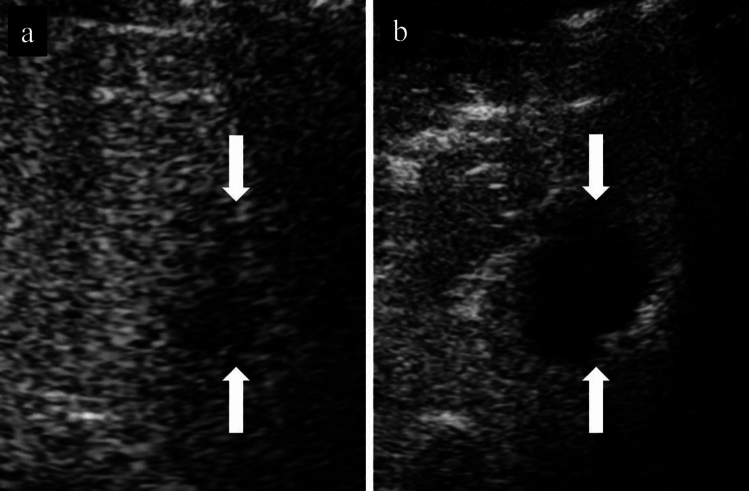
Assessment of response immediately after RFA using low mechanical index contrast imaging and high mechanical index contrast imaging. In the portal venous phase (**a**) of low mechanical index contrast imaging, it was difficult to evaluate contrast enhancement due to the background B-mode US and shielding by the ribs. After switching to high mechanical index contrast imaging (**b**), which is less affected by background B-mode US, it was possible to evaluate contrast enhancement at the ablation site by performing imaging while intermittently destroying the bubbles with frame rate 2. Contrast enhancement was not found at the RFA site in this case, indicating necrosis

**Fig. 18:**
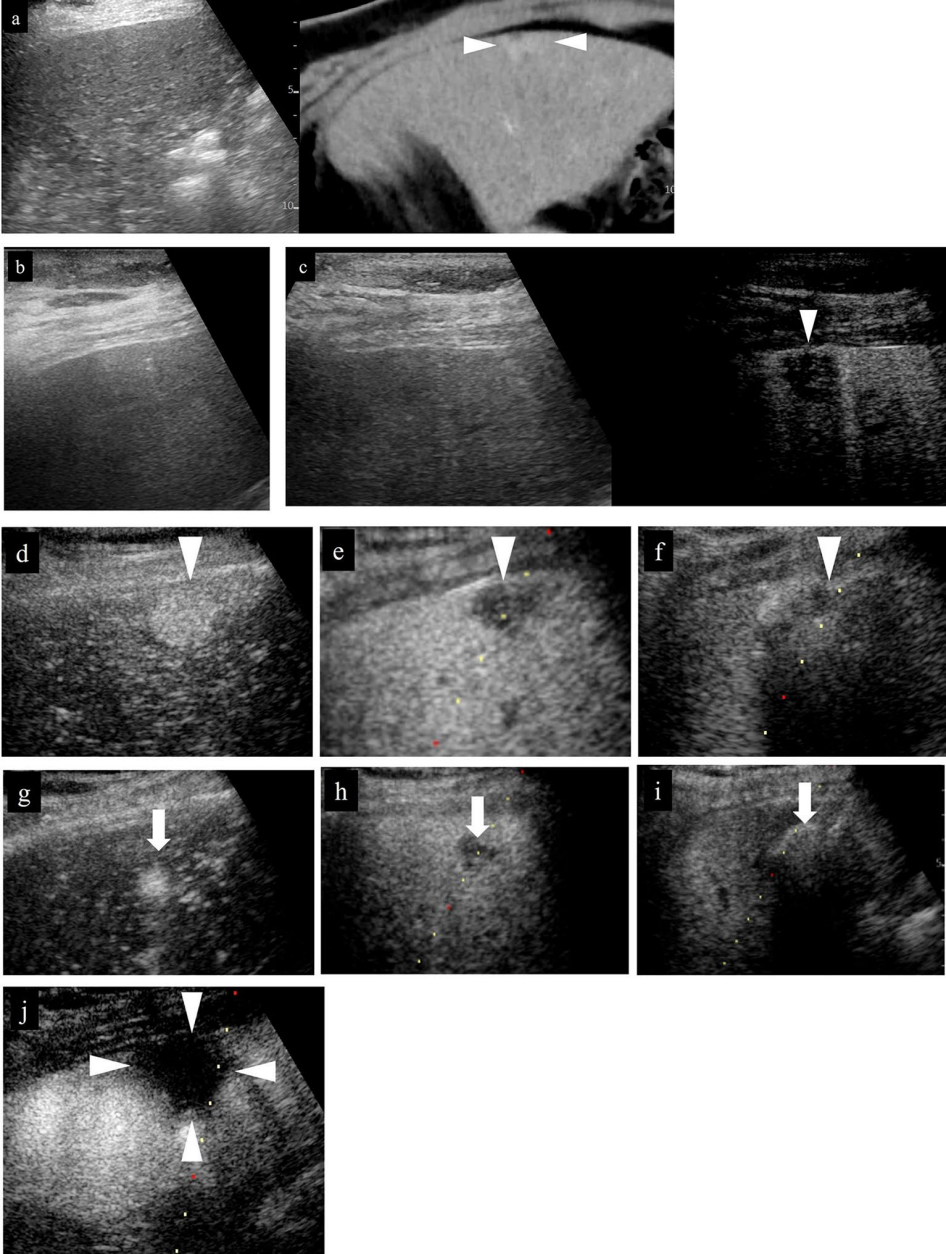

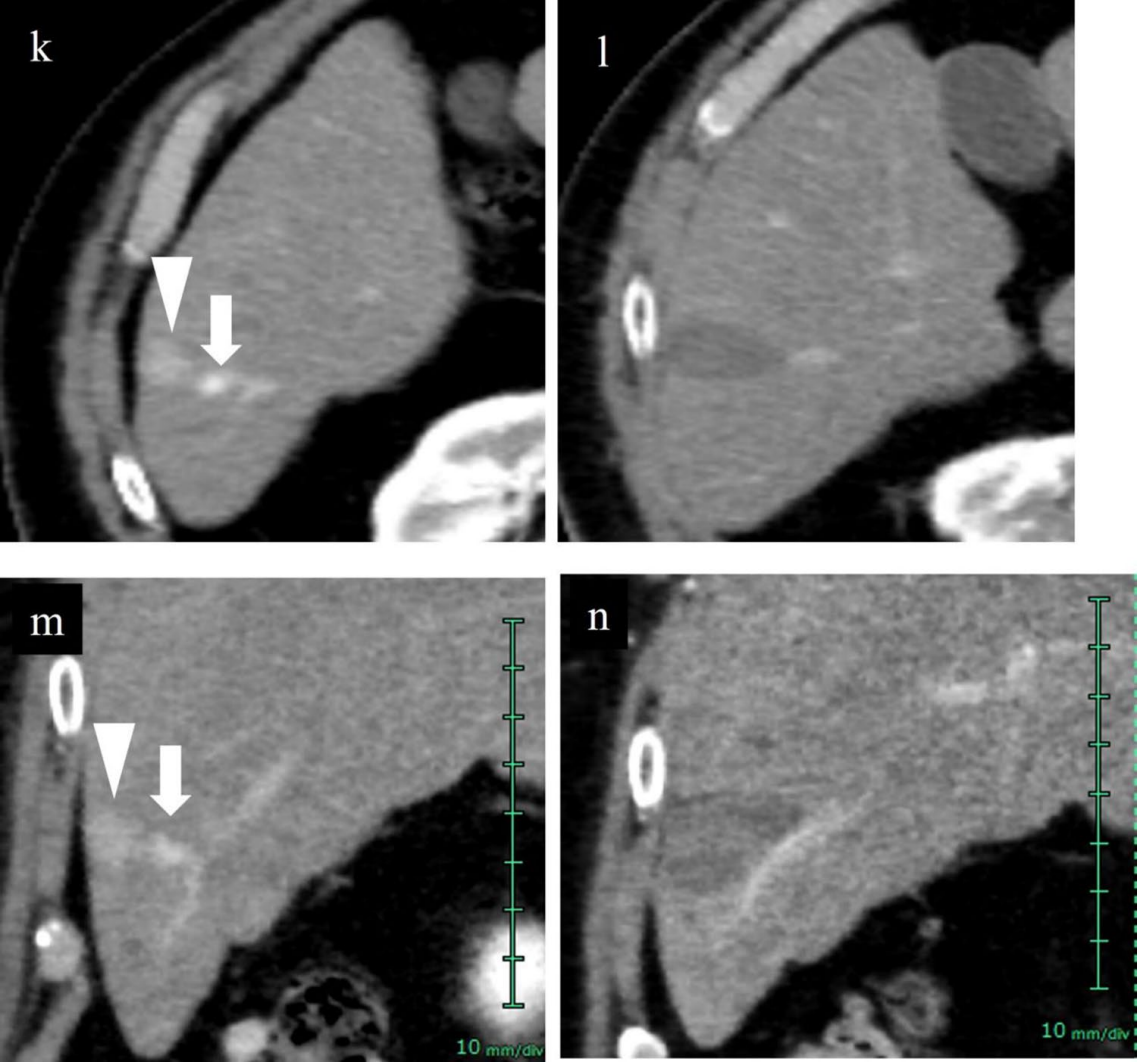
Hepatocellular carcinoma (right hepatic lobe posterior segment, tumor diameter 20 mm and 9 mm) that was difficult to detect on B-mode US. **a** On a fusion image of B-mode US with a convex probe and the arterial phase of contrast-enhanced CT, the tumor could not be detected at the site that corresponded to dense staining in the arterial phase of contrast-enhanced CT. **b** The lesion could also not be detected on B-mode US with a linear probe. **c** The lesion was depicted as hypoechoic in the post-vascular phase of low mechanical index contrast imaging. In addition to the liver surface, the lesion was seen in a slightly deep area in the arterial phase (**d**, **g**) and post-vascular phase (**e**, **h**) on low mechanical index contrast imaging on the day of RFA. RFA was performed for the respective target lesions in the post-vascular phase. Gas is seen in a more widespread area than the original lesion on B-mode US during RFA (**f**, **i**). **j** In the portal venous phase on high mechanical index contrast imaging performed after RFA, an area larger than the two lesions is observed as a non-contrast area. The axial section (**k**) and coronal section (**m**) in the arterial phase of contrast-enhanced CT before RFA, and the axial section (**l**) and coronal section (**n**) in the arterial phase of contrast-enhanced CT 1 month after RFA, indicate that the lesions were properly treated. However, we thought that caution was warranted as the axial section margin was less than 5 mm. Recurrence is not found 10 months after RFA. Arrowheads indicate the tumor border on the liver surface, and arrows show the tumor border in the slightly deep area

**Fig. 19:**
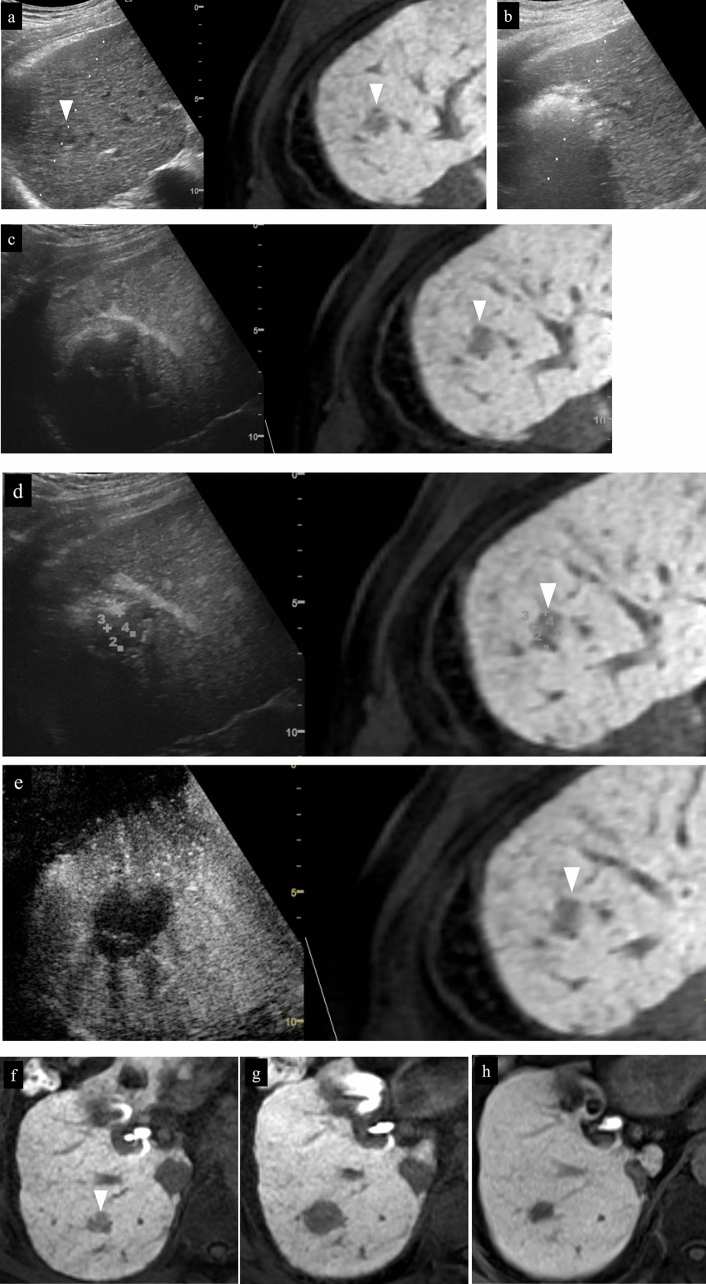
Hepatocellular carcinoma (right hepatic lobe posterior segment, tumor diameter 17 mm). **a** A circumscribed 17-mm hypointense lesion with an irregular contour is seen in the right lobe in the hepatobiliary phase on EOB-MRI. A circumscribed hypoechoic lesion is found on a fusion image with B-mode US. **b** RFA was performed at the same site. **c** Sonazoid contrast enhancement is not seen at the RFA site on a fusion image of the arterial phase of low mechanical index harmonic imaging immediately after RFA and the hepatobiliary phase of EOB-MRI before RFA. **d** By creating a fusion image of both using the enhanced portal vein and placing a GPS marker on the pre-RFA hepatobiliary phase of EOB-MRI, the GPS marker was depicted at the site corresponding to the tumor site in the portal venous phase of contrast-enhanced US immediately after RFA, and Sonazoid contrast enhancement was not found at the tumor site itself. On the other hand, it is difficult to evaluate the ablative margin due to the impact of gas and inflammation on the tumor body surface side. **e** Based on evaluation of the extent of necrosis on a fusion image comprised of the portal venous phase of high mechanical index contrast imaging and the hepatobiliary phase of EOB-MRI after RFA, it was determined that a sufficient margin was achieved. **f** A 17-mm hypointense lesion is seen in S7 in the hepatobiliary phase of EOB-MRI performed before treatment. **g** It was determined that the 17-mm lesion had been ablated in an area larger than that of the original lesion in the hepatobiliary phase of EOB-MRI performed after RFA treatment. **h** Local recurrence is not seen 3.5 years later. Arrowheads indicate the tumor border

**Fig. 20:**
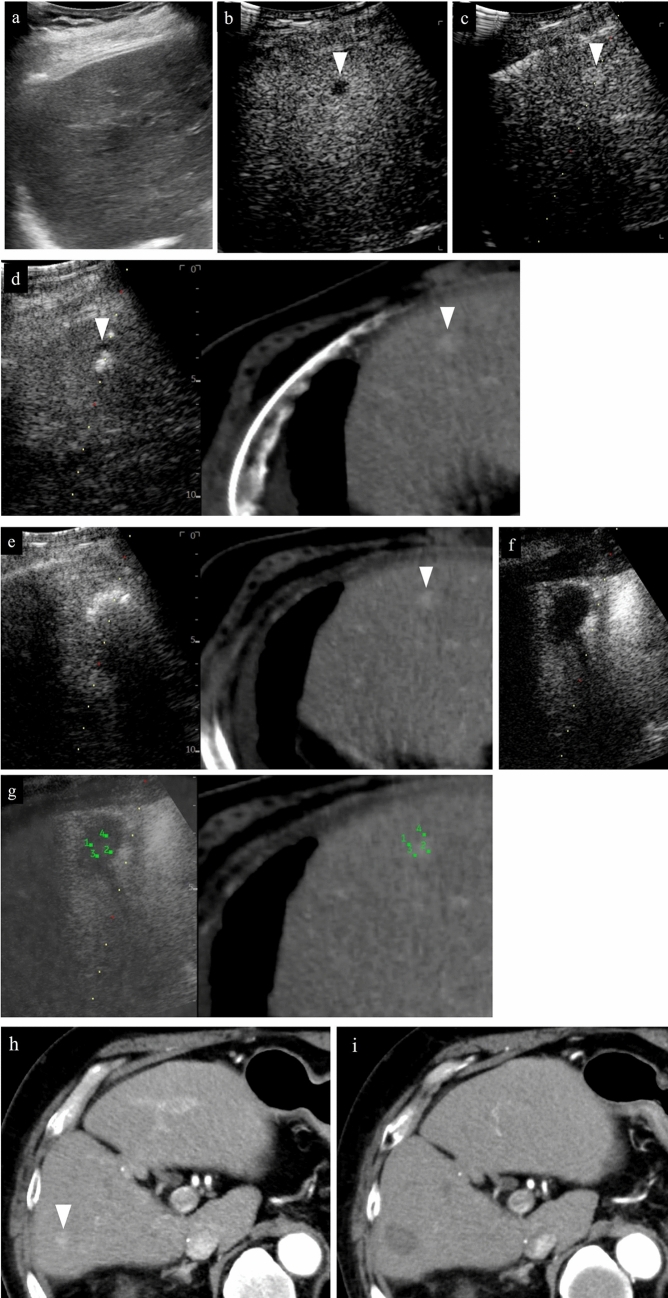
Hepatocellular carcinoma (right hepatic lobe anterior segment, tumor diameter 13 mm). **a** The lesion could not be detected on a fusion image of B-mode US and contrast-enhanced CT. **b** The lesion was depicted as hypoechoic in the post-vascular phase of low mechanical index contrast imaging. **c** The same site was densely stained after a second intravenous injection of Sonazoid and recognized as the target. **d** RFA was performed using a fusion image of the post-vascular phase of low mechanical index contrast imaging performed immediately after RFA (left screen) and the arterial phase of contrast-enhanced CT before RFA (right screen). The RFA needle pierces the lesion on the left screen. **e** Gas caused by RFA is seen. **f** Contrast enhancement is not seen at the RFA site immediately after RFA on the portal venous phase of high mechanical index contrast imaging. **g** By placing a GPS marker on the pre-RFA arterial phase of contrast-enhanced CT, the GPS marker was depicted at the site corresponding to the tumor site in the portal venous phase of contrast-enhanced US immediately after RFA, and absence of contrast enhancement at the RFA site was confirmed. **h** A 13-mm hypervascular lesion is seen in S8 in the arterial phase of contrast-enhanced CT performed before treatment. **i** It was determined that the 13-mm lesion had been ablated in an area larger than that of the original lesion in the arterial phase of contrast-enhanced CT performed after RFA. Arrowheads indicate the tumor border

Acoustic shadows are seen immediately after RFA as a result of steam gas caused by ablation, making visualization of the entire target lesion poor. Therefore, it is recommended that observation be performed again several minutes after ablation. Even if the acoustic shadow disappears, it is difficult to assess the ablative effect using low mechanical index contrast imaging as the lesion is visualized as hyperechoic. In such cases, performing high mechanical index contrast imaging will reduce the effects of the background B-mode US, allowing assessment of the presence or absence of bubbles flowing into the lesion (Fig. 17). Like typical HCC, the presence of residual HCC is generally suspected when there is dense staining in the arterial phase and washout in the post-vascular phase of contrast-enhanced US. Immediately after ablation, on the other hand, early dense staining is seen at the border of the ablated area in the arterial phase of contrast-enhanced US (Figs. 15, 16). The cause of this finding is believed to be an inflammatory response to the thermal injury caused by ablation. With respect to differentiation of inflammation from residual tumor, residual tumor is suspected when tissue is hypoenhancement in the post-vascular phase, and isoenhancement tissue is considered to be inflammation [39]. In addition, dense staining in the hypoenhanced area after re-administration of contrast agent should be assessed as residual tumor, and the margin should be ensured by performing additional ablation. Since the site of inflammation is often later found to be necrotic in the clinical setting, it is important to first confirm disappearance of the contrast effect in an area larger than the original tumor on contrast-enhanced US. Fusion imaging is sometimes used to confirm the position of the original tumor. High mechanical index contrast imaging, which is less impacted by high intensity due to steam gas, is effective for confirming the presence or absence of bubble inflow (Figs. 15, 16, 18, 19, 20).

### Detection of lesions on fusion imaging and assessment of treatment response

**Fig. 21:**
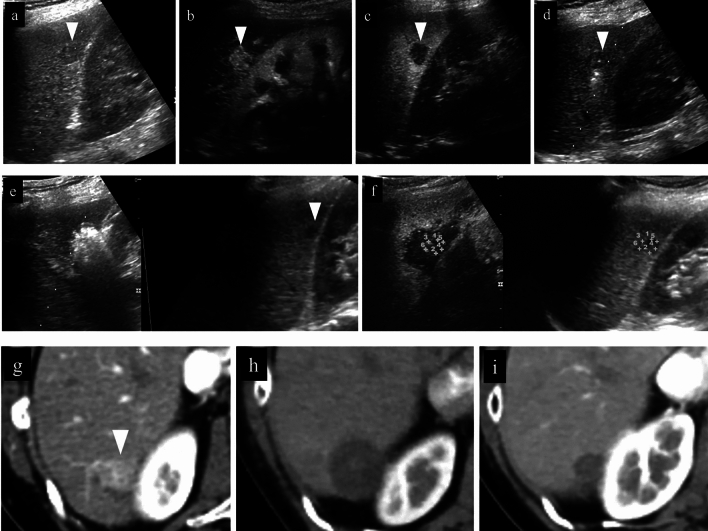
Hepatocellular carcinoma (right hepatic lobe posterior segment, tumor diameter 15 mm). **a** A 15-mm circumscribed, slightly hypoechoic lesion is seen in the posterior segment of the right lobe on B-mode US. **b** Dense staining in the arterial phase of low mechanical index harmonic imaging and washout in the portal venous phase 1.5 min after Sonazoid administration were seen (not shown). **c** It was hypoechoic in the post-vascular phase. **d** The deep part of the tumor was punctured with the extent of ablation set at 20 mm. **e** Monitoring was performed during ablation using a US-US fusion image that matched real-time B-mode US with B-mode spatial coordinates generated by acquiring 3D volume data immediately before that. An area larger than the original tumor was depicted as hyperechoic due to steam gas. **f** Shown here is a fusion image comprised of the portal venous phase of low mechanical index harmonic imaging immediately after RFA consisting of two punctures and the respective ablation and pre-RFA B-mode US. By placing a GPS marker on the pre-RFA B-mode US image, the GPS marker is depicted at the site corresponding to the tumor site in the portal venous phase of contrast-enhanced US immediately after RFA, as well. Given that contrast enhancement was not seen in an area larger than the target site, it was determined that appropriate treatment was achieved. The pathological diagnosis based on biopsy of the tumor performed immediately before RFA was poorly differentiated hepatocellular carcinoma. Based on a comparison of contrast-enhanced CT performed before and 1 month after treatment (**g**, **h**), it was determined that there was no residual tumor. **i** In the arterial phase of contrast-enhanced CT performed 4.5 years after RFA, shrinkage of the same site is seen, and recurrence is not found. Arrowheads indicate the tumor border

There is no issue with puncture of a lesion that can be clearly detected on B-mode US alone using fusion imaging (Fig. 19), but there are also lesions that are indistinct or cannot be visualized at all on B-mode US (Figs. 14, 15, 16, 18, 20). Performing contrast-enhanced US for such lesions not only allows us to diagnose the presence of the lesion and make a definitive diagnosis but also can be used for ablative therapy assistance and assessment of response to ablative therapy. In cases where the lesion cannot be identified on B-mode US, and cases where the original lesion cannot be identified due to steam gas associated with RFA or due to tissue degeneration caused by RFA itself, assess treatment response using a fusion image with pre-RFA contrast-enhanced CT, EOB-MRI, and contrast-enhanced US [40–42]. By creating a fusion image comprised of a post-RFA contrast-enhanced US image and a pre-RFA reference image and placing a GPS marker on pre-RFA B-mode US (Fig. 21), the arterial phase of contrast-enhanced CT (Fig. 20), and the hepatobiliary phase of EOB-MRI (Fig. 19), the GPS marker will be depicted at the site corresponding to the lesion site on the contrast-enhanced US image immediately after RFA. If the GPS marker represents the extent of the original lesion, we can confidently say that the original lesion was ablated if there is no contrast effect in an area larger than the GPS marker on contrast-enhanced US. It is possible to simultaneously evaluate the ablative margin, as well.

## Evaluation of complications

### Bleeding-related complications

**Fig. 22:**
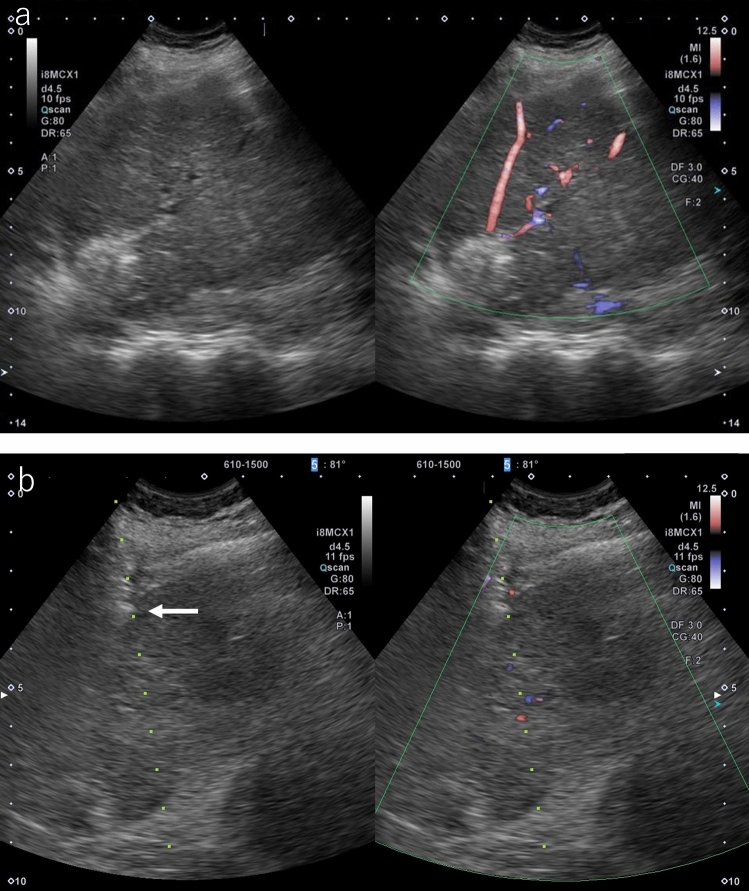
**a** A blood flow signal heading in the direction of the abdominal wall was seen on color Doppler US when the RFA needle was removed after ablation. **b** It was observed for several minutes, but compression hemostasis was performed by pressing down on the area around the hemorrhagic spot with the tip of the pneumoperitoneum needle as the blood flow signal did not wane (arrow)

Intraperitoneal bleeding: this occurs primarily as a result of bleeding from the liver surface. Carefully monitor the puncture route using color Doppler US when removing the puncture needle in order to evaluate the presence or absence of bleeding from the puncture route. The possibility of bleeding should be considered when a yellow to red warm color leading to the liver surface is displayed on the puncture line (Fig. 22). Hemostasis can usually be achieved via compression with the introducer or puncture needle, but if findings suggestive of bleeding continued to be observed, ablation of the puncture route is sometimes added.

Subcutaneous hematoma: this sometimes occurs along the puncture route. Bleeding can usually be stopped with local compression, but if it continues, CT or other examination should be performed under a suspicion of arterial injury, etc.

Hepatic subcapsular hematoma: this refers to hepatic subcapsular hematoma around the puncture site, and it is usually diagnosed based on diagnostic imaging performed the day after treatment. It sometimes results in severe anemia, and may require hemostasis with interventional radiology if bleeding continues.

**Fig. 23:**
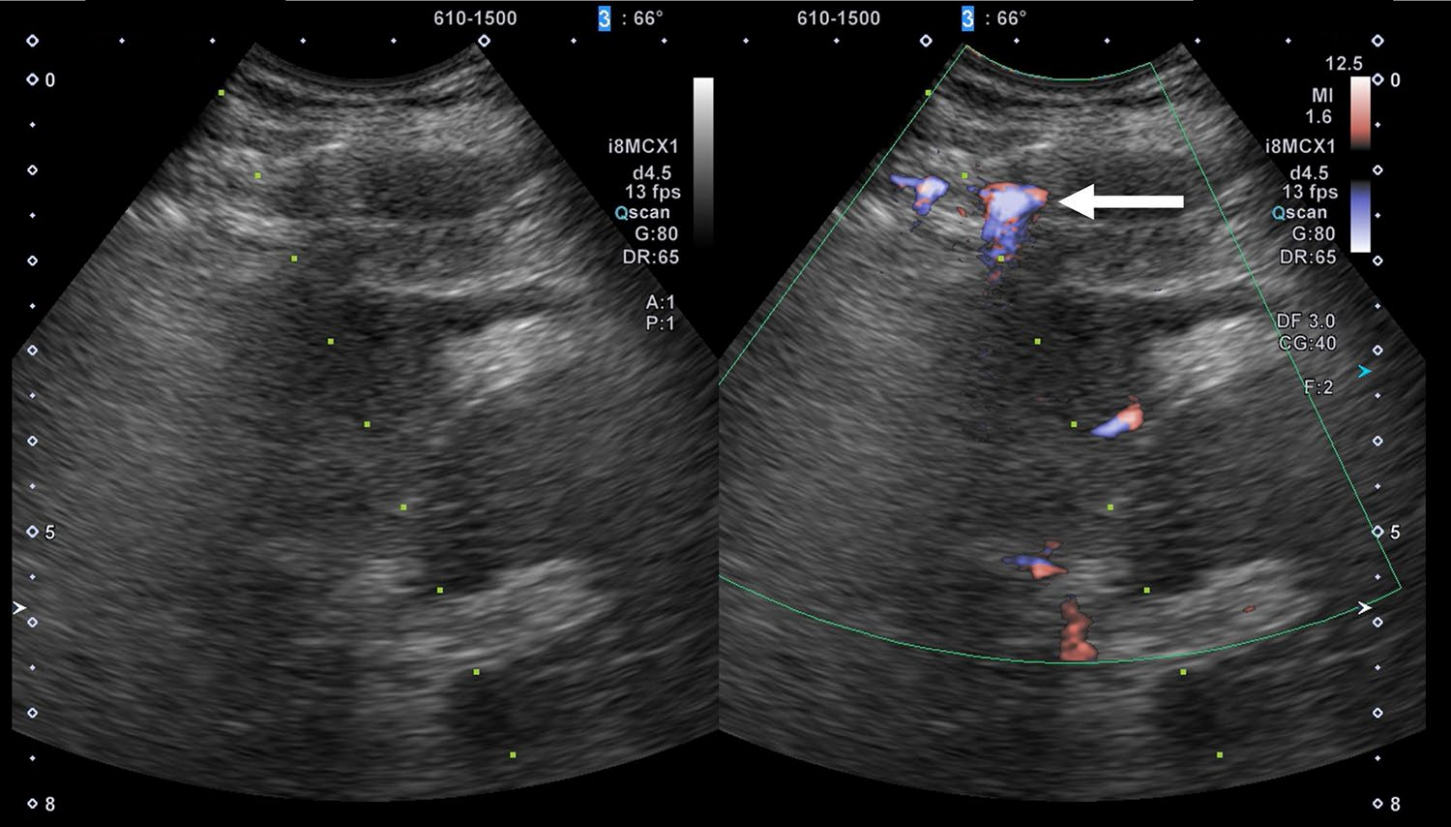
The puncture line was observed with color Doppler US during puncture of hepatocellular carcinoma located behind a rib in S4. A blood flow signal is seen near the rib (arrow), which may be an intercostal artery

Hemothorax: this occurs as a result of the puncture needle injuring intercostal arteries. Attention should also be paid to injury of the internal thoracic artery when treating a lesion in S4 of the liver (Fig. 23). Caution is required as the patient may fall into a state of shock with respiratory distress and decreased blood pressure within several hours after surgery. After ablation, not only bleeding from the liver surface but also the presence or absence of bleeding from intercostal arteries should be monitored with color Doppler US.

Hemobilia: injuring the wall of the hepatic artery with a puncture needle can cause a pseudoaneurysm. In a typical case, the pseudoaneurysm will rupture the adjacent bile duct a few days after the procedure, resulting in hemobilia. Since a pseudoaneurysm can be assessed using color Doppler US, blood flow along the puncture route should be carefully monitored if hemobilia is suspected [43].

### Portal vein/hepatic vein thrombosis

After ablation, blood clots may occur in the portal vein and hepatic veins near the target site. Evaluation of hepatic blood flow with color Doppler US during the postoperative course is extremely important from the standpoint of early detection of this complication.

### Gastrointestinal perforation

Perforation sometimes occurs when heat generated during ablation reaches the gastrointestinal tract when performing ablation of a lesion adjacent to the gastrointestinal tract. As such, one should always check whether the lesion is near the gastrointestinal tract using US, CT, or MRI before ablation. In addition, if performing contrast-enhanced US, evaluate the extent of the tumor and the presence or absence of non-tumor area around the tumor and the distance to the gastrointestinal tract. Perform ablative therapy using artificial ascites if the target lesion is touching the gastrointestinal tract or if the goal is total necrosis. If artificial ascites is not used, it will be necessary to adjust the time of ablation so that the ablative area does not become too large or have the patient skip taking meal on the day of ablation and follow the patient until the following morning.

### Pneumothorax

This complication can occur after ablation of a lesion near the lungs or as a result of creating artificial pleural effusion. If pneumothorax is strongly suspected, X-ray/CT should be promptly performed and appropriate action needs to be taken.

### Liver abscess

Although infrequent, this is an important complication of ablation. Liver abscess should be considered in the event of protracted fever and/or increased inflammatory response during the postoperative course. Liver abscess exhibits a variety of findings on contrast-enhanced US, but blood flow decreases and the diameter shrinks over time when inflammation improves.

### Hepatic infarction

Blood flow obstruction can occur and lead to tissue infarction in the same area as a result of injury to the dominant blood vessels (arteries and portal vein). It is often diagnosed at CT performed to assess treatment response on or after the day after treatment. A change in hepatic parenchyma echogenicity can sometimes be recognized on B-mode US, but it is usually diagnosed by evaluating blood-flow abnormalities using color Doppler or contrast-enhanced US.

### Biliary injury

This occurs in association with ablation of a tumor adjacent to Glisson's capsule. The bile duct at the injured site becomes stenosed, and the distal bile duct gradually dilates. The liver in the same area tends to atrophy in the long term. To prevent this complication, a drainage tube can be inserted in the bile duct beforehand, and ablation can be performed under perfusion with cooling water.

Thus, there are a variety of complications that can occur in association with ablation. Since many bleeding-related complications can be evaluated immediately after ablative therapy, the area surrounding the liver, the gallbladder, Morrison's pouch, and the area surrounding the diaphragm should be checked with B-mode US after ablation [44]. If Sonazoid-related contrast enhancement is found in pleural effusion or ascites on contrast-enhanced US, bleeding should be strongly suspected and action should be taken to identify the bleeding site. However, it is not necessarily easy to evaluate the liver as a whole or complications outside the liver with US alone; therefore, many institutions perform contrast-enhanced CT the day after treatment to not only evaluate the ablative effect but also investigate the presence or absence of complications.

## Conclusion

US is an excellent imaging support method for ablation of HCC, and it is an indispensable modality during the entire process from preoperative simulation to postoperative management. There have been a variety of advances related to ablation for HCC, including everything from indications and techniques to applicators and peripheral equipment. We hope that US will be utilized to facilitate safe and effective ablation based on the latest information and evidence presented here.
